# Evaluation of cytokine profiles related to *Mycobacterium tuberculosis* latent antigens using a whole-blood assay in the Philippines

**DOI:** 10.3389/fimmu.2024.1330796

**Published:** 2024-04-10

**Authors:** Ikkoh Yasuda, Naomi Ruth D. Saludar, Ana Ria Sayo, Shuichi Suzuki, Akira Yokoyama, Yuriko Ozeki, Haruka Kobayashi, Akihito Nishiyama, Sohkichi Matsumoto, Sharon E. Cox, Takeshi Tanaka, Yoshiro Yamashita

**Affiliations:** ^1^ Department of Clinical Medicine, Institute of Tropical Medicine, Nagasaki University, Nagasaki, Japan; ^2^ School of Tropical Medicine and Global Health, Nagasaki University, Nagasaki, Japan; ^3^ Department of General Internal Medicine and Clinical Infectious Diseases, Fukushima Medical University, Fukushima, Japan; ^4^ Department of General Internal Medicine and Infectious Diseases, Kita-Fukushima Medical Center, Fukushima, Japan; ^5^ San Lazaro Hospital, Manila, Philippines; ^6^ Department of Bacteriology, Niigata University Graduate School of Medicine, Niigata, Japan; ^7^ Department of Respiratory Medicine, Graduate School of Medicine, University of Tokyo, Tokyo, Japan; ^8^ Department of Medical Microbiology, Universitas Airlangga, Faculty of Medicine, Surabaya, Indonesia; ^9^ Division of Research Aids, Hokkaido University Institute for Vaccine Research & Development, Sapporo, Japan; ^10^ Department of Clinical Research, London School of Hygiene and Tropical Medicine, London, United Kingdom; ^11^ Department of Infectious Diseases, Nagasaki University Hospital, Nagasaki, Japan; ^12^ Infection Control and Education Center, Nagasaki University Hospital, Nagasaki, Japan; ^13^ Department of Respiratory Medicine, Shunkaikai Inoue Hospital, Nagasaki, Japan

**Keywords:** tuberculosis, latent tuberculosis infection, mycobacterial DNA-binding protein 1, α-crystallin, interferon-γ, tumor necrosis factor-α, interleukin-10, the Philippines

## Abstract

**Introduction:**

There is no useful method to discriminate between latent tuberculosis infection (LTBI) and active pulmonary tuberculosis (PTB). This study aimed to investigate the potential of cytokine profiles to discriminate between LTBI and active PTB using whole-blood stimulation with *Mycobacterium tuberculosis* (MTB) antigens, including latency-associated antigens.

**Materials and methods:**

Patients with active PTB, household contacts of active PTB patients and community exposure subjects were recruited in Manila, the Philippines. Peripheral blood was collected from the participants and used for whole-blood stimulation (WBS) with either the early secretory antigenic target and the 10-kDa culture filtrate protein (ESAT-6/CFP-10), Rv3879c or latency-associated MTB antigens, including mycobacterial DNA-binding protein 1 (MDP-1), α-crystallin (Acr) and heparin-binding hemagglutinin (HBHA). Multiple cytokine concentrations were analyzed using the Bio-Plex™ multiplex cytokine assay.

**Results:**

A total of 78 participants consisting of 15 active PTB patients, 48 household contacts and 15 community exposure subjects were eligible. The MDP-1-specific IFN-γ level in the active PTB group was significantly lower than that in the household contact group (p < 0.001) and the community exposure group (p < 0.001). The Acr-specific TNF-α and IL-10 levels in the active PTB group were significantly higher than those in the household contact (TNF-α; p = 0.001, IL-10; p = 0.001) and community exposure (TNF-α; p < 0.001, IL-10; p = 0.01) groups. However, there was no significant difference in the ESAT-6/CFP-10-specific IFN-γ levels among the groups.

**Conclusion:**

The patterns of cytokine profiles induced by latency-associated MTB antigens using WBS have the potential to discriminate between LTBI and active PTB. In particular, combinations of IFN-γ and MDP-1, TNF-α and Acr, and IL-10 and Acr are promising. This study provides the first demonstration of the utility of MDP-1-specific cytokine responses in WBS.

## 1 Introduction

Tuberculosis (TB) is one of the major causes of death worldwide, and 1.5 million people die from this infectious disease each year. Latent tuberculosis infection (LTBI) is defined as “a state of persistent immune response to stimulation by *Mycobacterium tuberculosis* (MTB) antigens with no evidence of clinically manifest active TB”, and up to one-third of the world’s population has LTBI (1). Most of MTB presumably exists in a dormant state in asymptomatic infected patients, but the bacterium may become activated and begin multiplying in 5% and 15% of infected individuals over a period of months to a few years (2, 3). LTBI is a major reservoir of TB, and the identification of individuals at high risk of progression from LTBI, coupled with immediate intervention at the early stage of progression, is crucial for disrupting MTB transmission. MTB infection spans a continuum, including intermediate stages such as ‘incipient’ and ‘subclinical’ tuberculosis between LTBI and active TB (4). For intervention at the early stage before onset, biomarkers are necessary to differentiate individuals at a high risk of progression or already undergoing progression from those in whom persistent MTB has been eliminated or is under control.

There is no gold standard direct microbiologic test for assessing LTBI, although the diagnostic gold standard for active TB is the detection of MTB by culture or nucleic acid amplification (NAA), including Xpert MTB/RIF and loop-mediated isothermal amplification (LAMP) assays (5–7). A tuberculin skin test (TST) or interferon-gamma releasing assay (IGRA) can substitute for LTBI testing; however, these assays are incapable of discriminating between LTBI and active TB or measuring progression to active TB, which creates problems when implementing TB elimination strategies in areas with high TB prevalence (8–11). Recently, functional signatures of T-cell responses have been comprehensively explored to monitor pathogenic viral and bacterial loads and disease activity by quantifying multiple cytokines (12). Although interferon-γ (IFN-γ) is assumed to play an essential role in the immune response to MTB, several other cytokines have been reported as candidates for biomarkers to discriminate between LTBI and active TB (13). Moreover, there has been growing interest in utilizing antigens specific to different stages of MTB infection (13). At the active TB state, the majority of MTB grow and express the early secretory antigenic target and the 10-kDa culture filtrate protein (ESAT-6/CFP-10), which are virulence factors that are lost in BCG (14) and mediate the translocation of MTB to the cytosol in infected myeloid cells (15). In contrast, dormant MTB expresses different types of protein antigens, such as alpha-crystallin-like protein (Acr), mycobacterial DNA-binding protein 1 (MDP-1) (16), and heparin-binding hemagglutinin (HBHA) (17). Acr is expressed under hypoxic conditions which mimics intracellular and intragranuloma conditions (18, 19), and is essential for the intracellular growth in macrophages (20). MDP-1 induces growth arrest of mycobacteria (21–23) and dormancy by causing DNA condensation by the MDP-1-specific structural functions (24). It also induces isoniazid tolerance (25, 26), which is a marker of dormant MTB (27). HBHA shows amino acid homology with MDP-1, also called laminin-binding protein (28), and shares functions as adhesins (17, 29). Immune responses against these antigens are reportedly useful for the discrimination of LTBI from active TB (30–35).

Globally, the largest number of new TB cases are reported in Southeast Asia, accounting for 45% of the total number of new cases worldwide in 2021. In addition, 7.0% of the total global incident cases in 2021 were reported in the Philippines, where the total estimated incidence of TB was 741,000, or 650 per 100,000 people (36). As a result of the large number of TB cases, LTBIs are also estimated to be prevalent in the Philippines (37).

This study aimed to investigate the potential of cytokine profiles to distinguish between LTBI and active TB using whole-blood stimulation (WBS) with MTB antigens, including MDP-1, a latency-associated antigen that has not been previously evaluated in WBS, in the Philippines, a high-TB prevalence setting.

## 2 Materials and methods

### 2.1 Study population

This study was a single-center, cross-sectional study. Patients were recruited from March 2019 to March 2020 at San Lazaro Hospital (SLH), a national infectious disease referral hospital located in Manila, the Philippines. The tertiary hospital has 500 beds and serves a poor local population in Metro Manila. We recruited patients with active pulmonary tuberculosis (PTB), household contacts of active PTB patients and community exposure subjects. The definition and enrollment criteria are described below.

- Active PTB: Patients who were newly diagnosed with PTB by direct smear test or NAA, including Xpert^®^ MTB/RIF (Cepheid, California, USA) and Loopamp™ MTBC detection kit (Eiken chemical, Tokyo, Japan) assays, before or within 5 days of initiating anti-TB treatment and without previous TB history.
- Household contacts: Subjects who were living in the same household for more than 2 months before diagnoses of index cases of bacteriologically confirmed active PTB and without any symptoms or previous TB history.
- Community exposure subjects: Subjects who were nonmedical employees at SLH without any symptoms, previous TB history or contact history with known TB cases.

Active PTB participants and household contacts were enrolled from the Starting Anti-Tuberculosis Treatment (St-ATT) Cohort. ISRCTN16347615 (38).

We excluded people younger than 18 years old and those with conditions that may affect the immune response, including pregnancy, lactation, known HIV infection, diabetes mellitus and/or autoimmune diseases. We also excluded subjects for whom written informed consent was not obtained.

### 2.2 Data collection

Demographic and clinical information, including age, sex, body mass index (BMI), past medical history and comorbidities, and symptoms of and diagnostic methods for active PTB, was obtained by hospital and research staff. A total of 3 mL of peripheral blood from the participants was collected in a single blood collection tube containing lithium heparin as an anticoagulant by venipuncture, and this blood was used for whole-blood stimulation and complete blood count tests.

### 2.3 Whole-blood stimulation and multiplex assays

Whole blood was mixed with the same amount of RPMI 1640 (Wako Junyaku Co., Tokyo, Japan) and plated in 300-µl aliquots into each well of a 96-well round-bottom culture plate. For stimulation, either ESAT-6/CFP-10 (each at a concentration of 5 µg/mL), MDP-1 (10 µg/mL), Acr (5 µg/mL), HBHA (5 µg/mL), Rv3879c (5 µg/mL), or concanavalin A (35 µg/mL; positive control) or no stimulant (negative control) was added to each well. Recombinant ESAT-6/CFP-10 and Acr were expressed in ClearColi BL21(DE3) cells using previously created expression plasmids (39). For expression of Rv3879c, the coding sequence (CDS) with the addition of an NdeI site at the N-terminus and 6XHistidine and a HindIII site at the C-terminus was synthesized (**Supplementary Figure 1**) and inserted into the Nde1 and HindIII sites of pET22b(+). This construct was then introduced into ClearColi BL21(DE3). Recombinant MTB proteins, such as ESAT-6/CFP-10, Acr, and Rv3879c, were expressed by addition of IPTG and purified by a previously described method (32). pSO-AMI-MDP1_Mtb_ (23) was used to express MTB MDP-1 as a 6xHIS-tagged protein in *M. smegmatis*. For expression of HBHA, the CDS of HBHA with the addition of an NdeI site at the N-terminus and 6XHistidine and Kpn1 sites at the C-terminus was synthesized (**Supplementary Figure 1**), inserted into pSO-AMI and introduced into *M. smegmatis*. Recombinant *M. smegmatis* strains were incubated on Sauton media containing 10 µg/mL kanamycin; expression of recombinant MTB MDP-1 and HBHA was induced by addition of acetoamide at a final concentration of 0.2% and purified by His GraviTrap by a described method (40). All antigen stimulations were performed in the presence of anti-CD28 and anti-CD49d costimulators (0.3 µg/mL). (eBioscience, USA) After incubation at 37°C with atmospheric concentrations of carbon dioxide (approximately 0.04%) for 22 hours, the supernatant was collected and immediately frozen at -80°C. The frozen supernatant samples were transported to the Institute of Tropical Medicine in Nagasaki, Japan, on dry ice. The concentrations of 10 cytokines, IFN-γ, TNF-α (tumor necrosis factor-α), IL (interleukin)-2, IL-5, IL-9, IL-10, IL-13, IL-17, IL-22 and IL-27, were analyzed using a 10-plex Bio-Plex Pro™ Human Th17 Magnetic Bead Panel (EMD Millipore Corporation, Billerica, MA, USA) according to the manufacturer’s instructions. These signature cytokines were comprehensively selected to represent known T helper cell subsets, including Th1, Th2, Treg, Th17, Th9, Th22 and Tr27 (41, 42). The Bio-Plex 200 system and Bio-Plex manager software (version 5.0, Bio-Rad, USA) were used to read the panels. All samples were assayed in duplicate. The details of each MTB antigen were described previously (16, 17, 19, 43). Cytokine responses were defined as the difference between the cytokine concentration for the antigen of interest and the cytokine concentration for the negative control to adjust for nonspecific responses.

### 2.4 Endpoint

The primary endpoint was the concentration of each of the 10 cytokines released by WBS against the MTB-related antigens.

### 2.5 Statistical analysis

All clinical information and blood analysis data were recorded into an electronic database and analyzed. Comparisons of continuous variables, including age, BMI, CBC count and concentrations of each cytokine among the active PTB, household contact and community exposure subjects, were performed using the Kruskal-Wallis test followed by Dunn’s *post hoc* test with Holm adjustment. Pairwise comparisons were performed using the Mann-Whitney U test. We used linear regression to adjust for the potential confounders age, sex and BMI. A p value less than 0.05 after adjustment was considered to indicate statistical significance. Receiver operating characteristic (ROC) curve analysis was performed to evaluate the discriminatory performance of MTB antigen-specific cytokines. All analyses were performed with Stata version 18.0 (Stata Corp., College Station, TX, USA).

### 2.6 Ethical issues

This study was approved by the Research Ethical and Review Unit of San Lazaro Hospital, the Philippines (number: SLH-RERU-2018-005-E), and the Institutional Review Board of the School of Tropical Medicine and Global Health, Nagasaki University, Japan (number: NU_TMGH_2019-050-3). The study was fully explained to all participants in Filipino and English, and written informed consent was obtained from all the participants. Anonymized data were used for analysis.

## 3 Results

### 3.1 Basic characteristics

**Table 1** shows the basic characteristics of the participants. A total of 78 participants consisting of 15 participants with active PTB, 48 household contacts and 15 community exposure subjects were eligible. The median ages were 36.0 years (range: 19.0–49.0) in the active PTB group, 47.0 years (range: 18.0–79.0) in the household contact group and 33.0 years (range: 22.0–55.0) in the community exposure group. A total of 11 (73.3%) active PTB participants were diagnosed by NAA; the other 4 (26.7%) active PTB participants were diagnosed by direct smear microscopy. All active PTB participants were given anti-TB treatment, and the median duration since starting treatment was 2 days (range: 0–5). A total of 42 (87.5%) index cases of the household contacts were diagnosed by NAA; the other 6 (12.5%) index cases of the household contacts were diagnosed by direct smear microscopy. The comorbidities of the household contacts included hypertension (2 participants) and hepatitis B (1 participant). Active PTB patients were more likely to be male and have a low BMI.

**Table 1:** Basic characteristics of the participants.

		Active PTB (n=15)	Household contacts (n=48)	Community exposure subjects (n=15)	p value
Age	year				
	median. (range)	36.0 (19.0–49.0)	47.0 (18.0–79.0)	33.0 (22.0–55.0)	0.031 ‡
Female	no. (%)	2 (13.3)	34 (70.8)	8 (53.3)	<0.001 †
BMI	kg/m^2^				
	median. (range)	18.7 (14.1–23.3)	24.1 (17.0–37.6)	25 (19.1–39.0)	<0.001 ‡
Comorbidity	no. (%)	0 (0.0)	2 (4.2)	0 (0.0)	1 †
Diagnostic method	no. (%)	for participant	for index case		
NAA		11 (73.3)	42 (87.5)	NA	
Only smear		4 (26.7)	6 (12.5)	NA	
CBC count	median. (range)				
WBC	10^3^/µl	9.1 (6.5–13.4)	7.2 (3.8–12.5)	8.8 (4.3–13.3)	0.002 ‡
Neutrophils	10^3^/µl	6.3 (2.9–10.8)	3.8 (2.0–7.2)	4.9 (2.3–8.6)	<0.001 ‡
	%	68.0 (31.8–82.1)	53.7 (39.1–68.8)	56.1 (46.9–64.6)	<0.001 ‡
Lymphocytes	10^3^/µl	1.6 (1.0–3.0)	2.5 (0.7–4.6)	2.9 (1.5–4.3)	<0.001 ‡
	%	18.5 (8.4–32.7)	35.3 (19.5–50.7)	32.8 (24.3–42.4)	<0.001 ‡
Monocytes	10^3^/µl	0.7 (0.4–1.3)	0.5 (0.2–0.9)	0.6 (0.3–0.8)	<0.001 ‡
	%	8.2 (4.5–11.2)	6.7 (4.1–8.8)	7.1 (5.0–9.3)	0.019 ‡
Eosinophils	10^3^/µl	0.43 (0.00–2.76)	0.29 (0.03–1.11)	0.33 (0.07–0.69)	0.553 ‡
	%	5.0 (0.0–30.3)	4.0 (0.4–12.4)	3.8 (1.0–8.3)	0.744 ‡

For comparisons among the three groups, † Fisher’s exact test for categorical variables or ‡ the Kruskal-Wallis test followed by Dunn’s *post hoc* test with Holm adjustment for continuous variables were used.

### 3.2 Cytokine response to ESAT-6/CFP-10

**Figure 1:**
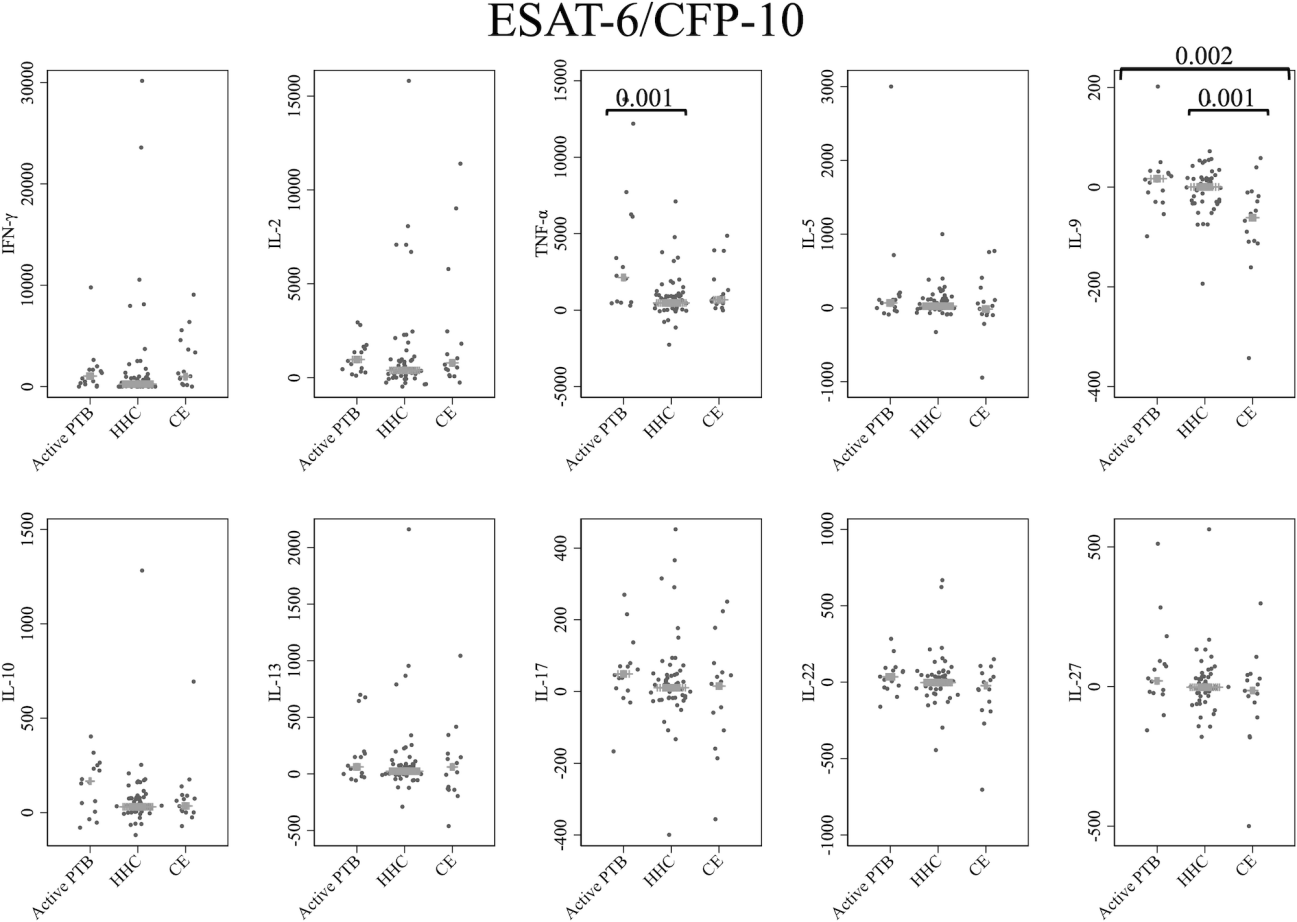
Cytokine response to ESAT-6/CFP-10. The X-axis shows the participant groups. The Y-axis shows the concentrations of cytokines (pg/mL for IFN-γ, IL-2, TNF-α, IL-5, IL-9, IL-10, IL-13 and IL-17; ng/mL for IL-22 and IL-27). The horizontal lines show the medians. The numbers in the figure indicate adjusted p values. Comparisons of the concentrations of each cytokine among active PTB patients, household contacts and community exposures were performed using the Kruskal-Wallis test followed by Dunn’s *post hoc* test with Holm adjustment. We used linear regression to adjust for the potential confounders age, sex and BMI. Active PTB, active pulmonary tuberculosis; HHC, household contact; CE, community exposure.

**Figure 1** shows the cytokine responses to ESAT-6/CFP-10. There was no significant difference in IFN-γ levels among the groups. In contrast, the TNF-α level in the active PTB group was significantly higher than that in the household contact group (p = 0.001); the IL-9 level in the community exposure group was significantly lower than that in the active PTB (p = 0.002) and household contact (p = 0.001) groups. However, no cytokines were identified that could significantly distinguish the active PTB group from both the household contact group and the community exposure group in response to ESAT-6/CFP-10 stimulation.

### 3.3 Cytokine response to MDP-1

**Figure 2:**
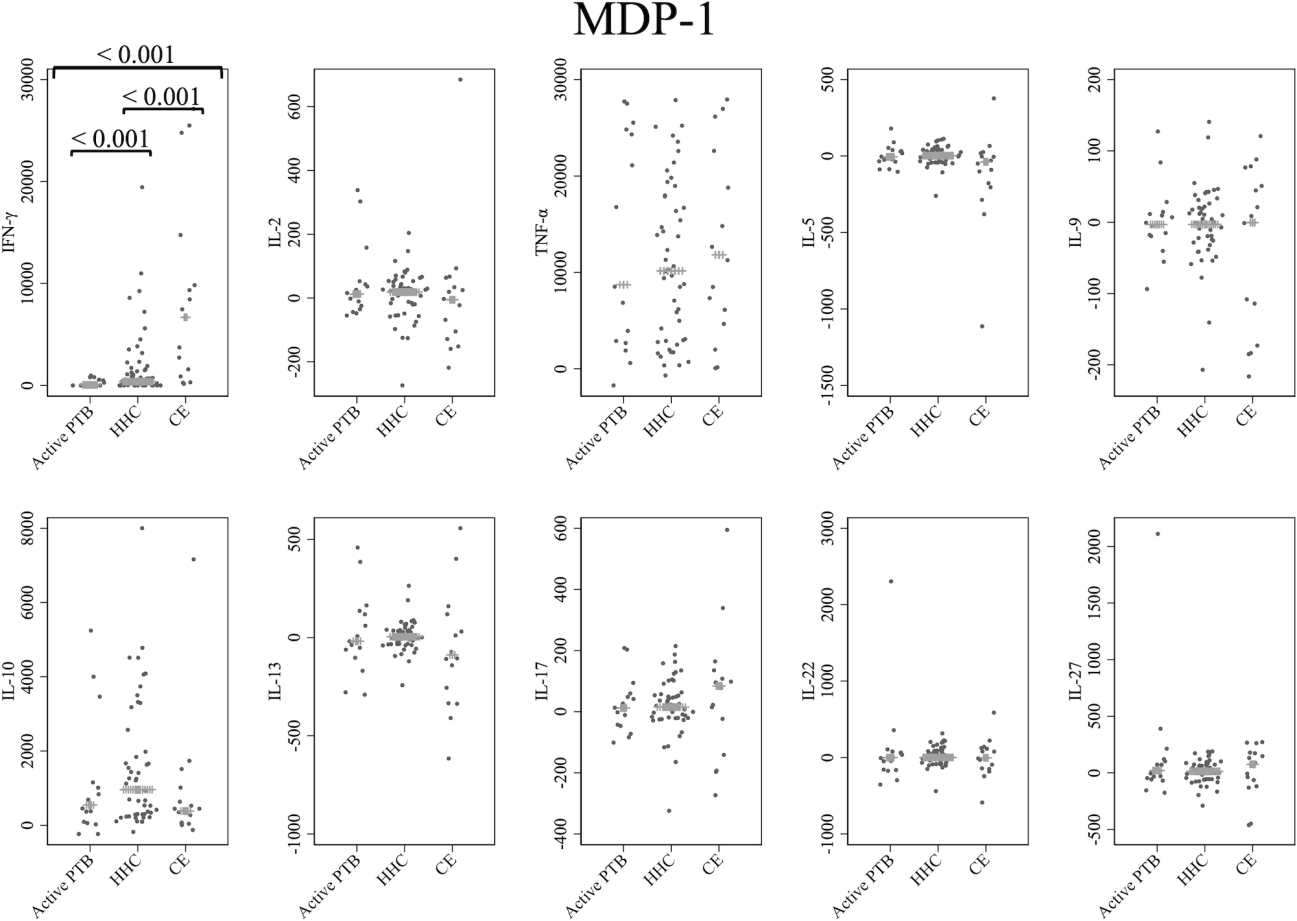
Cytokine response to MDP-1. The X-axis shows the participant groups. The Y-axis shows the concentrations of cytokines (pg/mL for IFN-γ, IL-2, TNF-α, IL-5, IL-9, IL-10, IL-13 and IL-17; ng/mL for IL-22 and IL-27). The horizontal lines show the medians. The numbers in the figure indicate adjusted p values. Comparisons of the concentrations of each cytokine among active PTB patients, household contacts and community exposures were performed using the Kruskal-Wallis test followed by Dunn’s *post hoc* test with Holm adjustment. We used linear regression to adjust for the potential confounders age, sex and BMI. Active PTB, active pulmonary tuberculosis; HHC, household contact; CE, community exposure.

Cytokine responses to MDP-1 are provided in **Figure 2**. The IFN-γ level in the active PTB group was significantly lower than that in the household contact group (p < 0.001) and the community exposure group (p < 0.001). The IFN-γ level in the household contact group was significantly lower than that in the community exposure group (p < 0.001). Specifically, MDP-1-specific IFN-γ demonstrated the ability to detect all differences between the active PTB and household contact groups, between the active PTB and community exposure groups, and between the household contact and community exposure groups.

### 3.4 Cytokine response to Acr

**Figure 3:**
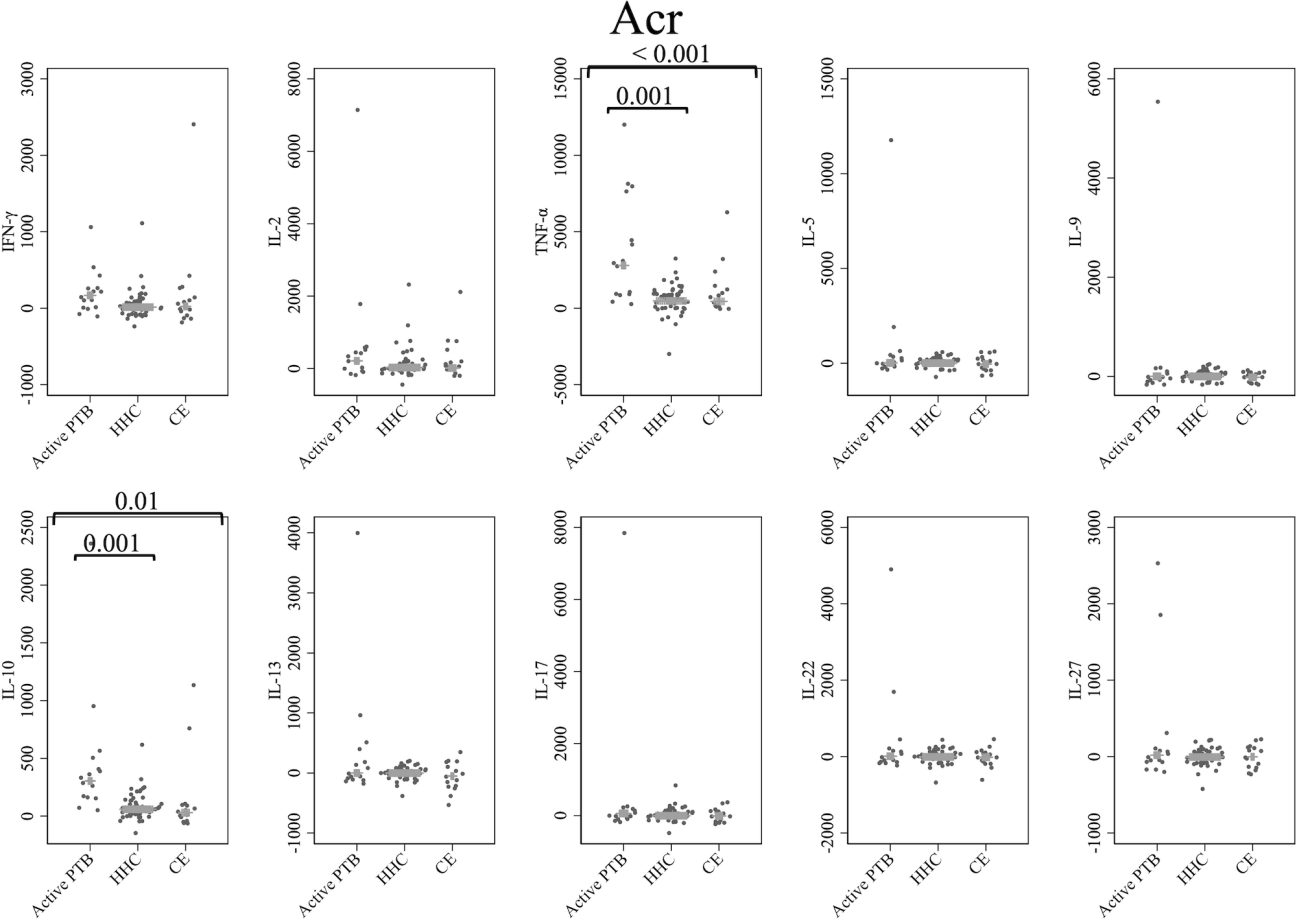
Cytokine response to Acr. The X-axis shows the participant groups. The Y-axis shows the concentrations of cytokines (pg/mL for IFN-γ, IL-2, TNF-α, IL-5, IL-9, IL-10, IL-13 and IL-17; ng/mL for IL-22 and IL-27). The horizontal lines show the medians. The numbers in the figure indicate adjusted p values. Comparisons of the concentrations of each cytokine among active PTB patients, household contacts and community exposures were performed using the Kruskal-Wallis test followed by Dunn’s *post hoc* test with Holm adjustment. We used linear regression to adjust for the potential confounders age, sex and BMI. Active PTB, active pulmonary tuberculosis; HHC, household contact; CE, community exposure.

**Figure 3** shows the cytokine responses to Acr. The TNF-α and IL-10 levels in the active PTB group were significantly higher than those in both the household contact (TNF-α; p = 0.001, IL-10; p = 0.001) and community exposure (TNF-α; p < 0.001, IL-10; p = 0.01) groups. Specifically, Acr-specific TNF-α and Acr-specific IL-10 could significantly distinguish the active PTB group from both the household contact group and the community exposure group.

### 3.5 Cytokine response to HBHA

**Figure 4:**
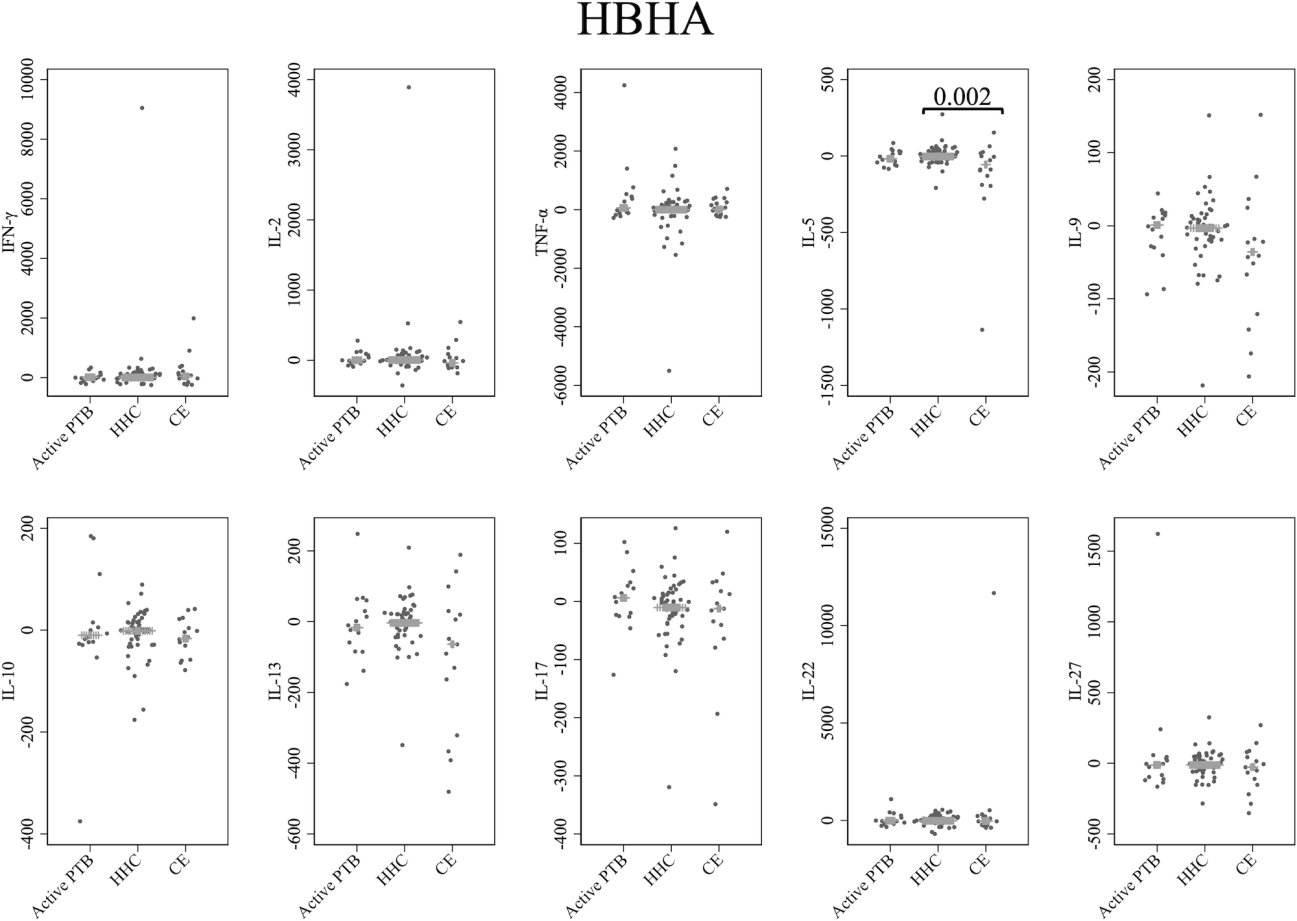
Cytokine response to HBHA. The X-axis shows the participant groups. The Y-axis shows the concentrations of cytokines (pg/mL for IFN-γ, IL-2, TNF-α, IL-5, IL-9, IL-10, IL-13 and IL-17; ng/mL for IL-22 and IL-27). The horizontal lines show the medians. The numbers in the figure indicate adjusted p values. Comparisons of the concentrations of each cytokine among active PTB patients, household contacts and community exposures were performed using the Kruskal-Wallis test followed by Dunn’s *post hoc* test with Holm adjustment. We used linear regression to adjust for the potential confounders age, sex and BMI. Active PTB, active pulmonary tuberculosis; HHC, household contact; CE, community exposure.

Cytokine responses to HBHA are illustrated in **Figure 4**. The IL-5 level in the community exposure group was significantly lower than that in the household contact group (p = 0.002). However, no cytokines were identified that could significantly distinguish the active PTB group from both the household contact group and the community exposure group in response to HBHA stimulation.

### 3.6 Cytokine response to Rv3879c

**Figure 5:**
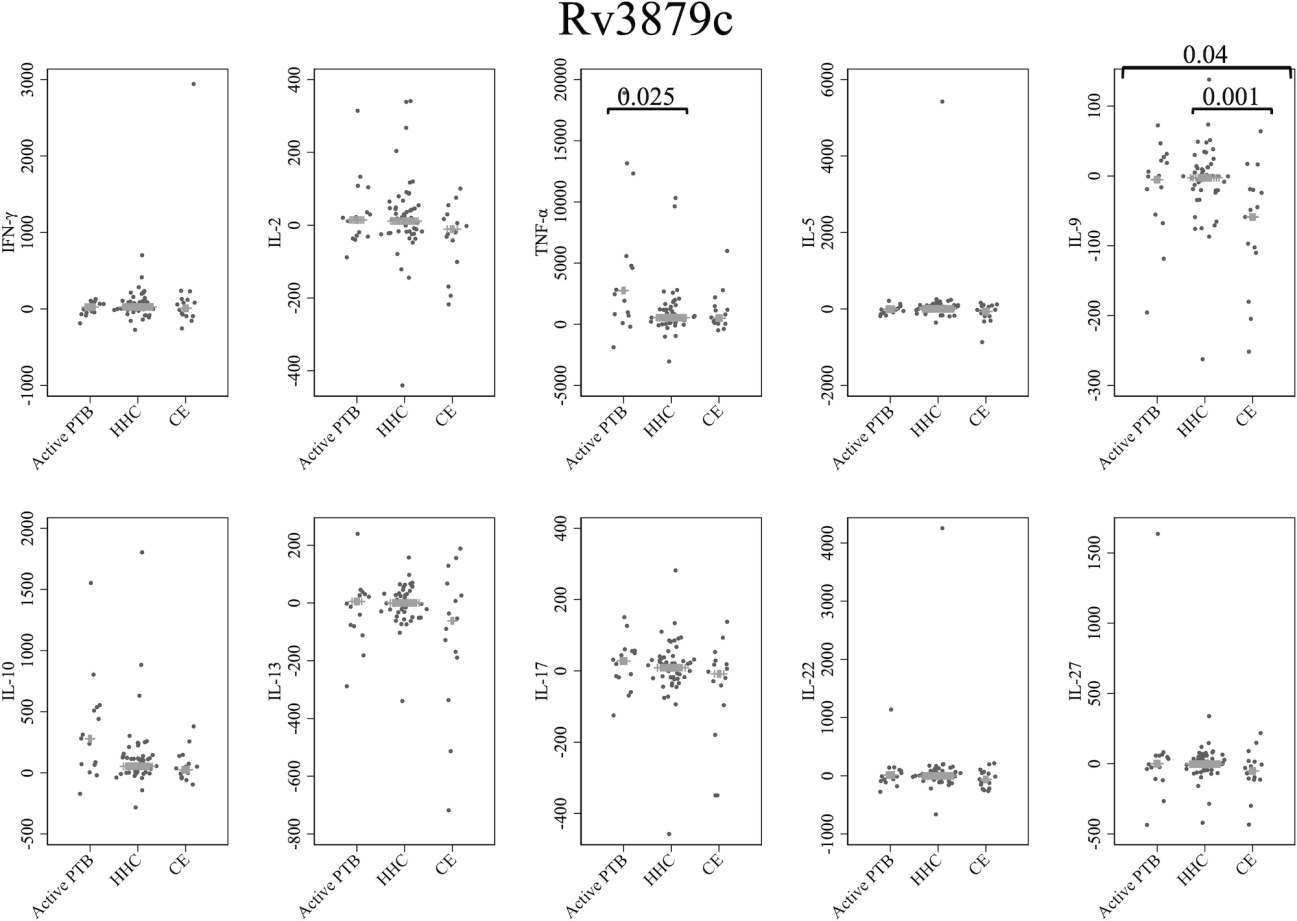
Cytokine response to Rv3879c. The X-axis shows the participant groups. The Y-axis shows the concentrations of cytokines (pg/mL for IFN-γ, IL-2, TNF-α, IL-5, IL-9, IL-10, IL-13 and IL-17; ng/mL for IL-22 and IL-27). The horizontal lines show the medians. The numbers in the figure indicate adjusted p values. Comparisons of the concentrations of each cytokine among active PTB patients, household contacts and community exposures were performed using the Kruskal-Wallis test followed by Dunn’s *post hoc* test with Holm adjustment. We used linear regression to adjust for the potential confounders age, sex and BMI. Active PTB, active pulmonary tuberculosis; HHC, household contact; CE, community exposure.

**Figure 5** presents cytokine responses to Rv3879c. The TNF-α level in the active PTB group was significantly higher than that in the household contact group (p = 0.025). The IL-9 level in the community exposure group was significantly lower than that in the active PTB group (p = 0.04) and the household contact group (p = 0.001). However, no cytokines were identified that could significantly distinguish the active PTB group from both the household contact group and the community exposure group in response to Rv3879c stimulation.

**Supplementary Figure 2** shows the box and whisker plots of cytokine responses without outside values for each antigen.

### 3.7 Assessment of discriminatory performance

These results suggested the ability of the five MTB antigen-specific cytokines to distinguish active PTB patients from household contact subjects. These cytokines included MDP-1-specific IFN-γ, Acr-specific TNF-α, Acr-specific IL-10, ESAT-6/CFP-10-specific TNF-α, and Rv3879c-specific TNF-α. Specifically, the first three, namely, MDP-1-specific IFN-γ, Acr-specific TNF-α, and Acr-specific IL-10, were able to distinguish active PTB from both the household contact subjects and the community exposure subjects. ROC curve analysis was also conducted to assess the discriminatory performance of these MTB antigen-specific cytokines for distinguishing among active PTB patients, household contacts and community exposure subjects. As demonstrated in **Supplementary Figure 3**, the highest area under the ROC curve (AUC) was calculated for Acr-specific IL-10, followed by Acr-specific TNF-α, in distinguishing between active PTB and household contacts (AUC = 0.90 and 0.88, respectively). Additionally, MDP-1-specific IFN-γ exhibited the highest AUC in discriminating between active PTB patients and community exposure subjects (AUC = 0.87) and between household contacts and community exposure subjects (AUC = 0.75).

### 3.8 Comparison between two groups: “active PTB” group versus group of “household contacts and community exposure subjects”

From the perspective of individuals with a high probability of nonactive TB infection, the household contact group and community exposure group were combined and analyzed as a single group. **Supplementary Table 1** shows the comparison of basic characteristics, and **Supplementary Figure 4** shows the comparison of cytokine responses to each antigen between the two groups: “active PTB” group versus the group of “household contacts and community exposure subjects”. The cytokines that showed significant differences between the two groups were IFN-γ when stimulated by MDP-1; IL-2, TNF-α and IL-10 when stimulated by Acr; TNF-α when stimulated by ESAT-6/CFP-10; and TNF-α when stimulated by Rv3879c.

## 4 Discussion

This was an exploratory study to document the patterns of cytokine profiles induced by MTB antigens, including latency-associated antigens, among three groups, active PTB, household contacts and community exposure subjects, using WBS in the Philippines. Certain combinations of latency-associated MTB antigens showed significant differences in cytokine levels among the groups. Specifically, the three MTB antigen-specific cytokines, namely, MDP-1-specific IFN-γ, Acr-specific TNF-α, and Acr-specific IL-10, could distinguish active PTB patients from both the household contact subjects and the community exposure subjects, and they exhibited high discriminatory performance in the ROC curve analysis. The level of IFN-γ induced by MDP-1 was significantly lower in the active PTB group than in both the household contact group and the community exposure group. The TNF-α and IL-10 levels induced by Acr were significantly higher in the active PTB group than in the other two groups. The same combinations described above also led to significantly different cytokine levels between the “active PTB” group and the group of “household contacts and community exposure subjects”, as shown in **Supplementary Figure 4**. The current study suggested the potential of the following cytokine-stimulating antigen combinations for differentiating LTBI from active TB when using WBS: IFN-γ and MDP-1, TNF-α and Acr, and IL-10 and Acr.

In the present study, the IFN-γ levels induced by ESAT-6/CFP-10 were not significantly different among the three groups. Because IGRAs are methods for assessing IFN-γ induced by ESAT-6/CFP-10, this result indicates the limitations of IGRAs in countries with a high TB burden. In most cases, TB develops shortly after MTB infection, and a distinction is generally made between recent LTBI (infection occurring within two years), which is associated the greatest risk of developing the disease, and remote LTBI (infection occurring more than two years ago), which is related to a reduced risk of developing the disease (44, 45). However, IGRAs cannot distinguish between LTBI and active TB or between recent LTBI and remote LTBI. In countries with a low TB incidence, systematic testing with IGRAs is recommended for populations at risk, and individuals who test positive through IGRA-based screening are generally offered preventive treatment as a part of the TB elimination strategy (46). In areas with a high prevalence of active TB and remote LTBI, however, IGRAs that cannot differentiate recent LTBI have poor predictive values for TB progression, which is a hindrance to TB elimination (47, 48). The Philippines is a country with a high incidence of active TB and LTBI. The crude rate of LTBI in the Philippines was 37.12% in 2019, based on data from the Global Health Data Exchange (GHDx) query tool (37, 49), and a study conducted in 10 tertiary hospitals in the Philippines reported a prevalence of LTBI among healthcare workers of 84.87% (50). Although there was uncertainty due to the inability to use IGRAs for the diagnosis of LTBI in this study, it may be considered that the household contact group represented recent LTBI resulting from new TB exposure, and the community exposure group, who comprised employees at the tertiary hospital, represented remote LTBI derived from a high LTBI prevalence population in the Philippines. Thus, the findings regarding ESAT-6/CFP-10-specific IFN-γ in the current study are compatible with the limited performance of IGRAs in differentiating among active TB, recent LTBI and remote LTBI, and in identifying individuals at high risk of developing TB.

In contrast, MDP-1-specific IFN-γ, Acr-specific TNF-α and Acr-specific IL-10 led to significant differences in cytokine levels both between the active PTB patients and household contact subjects and between the active PTB patients and community exposure subjects. IFN-γ and TNF-α are known proinflammatory cytokines involved in the Th1 immune response and play important roles in host defense against MTB infection; in contrast, IL-10 is involved in the non-Th1 immune response and is known as an anti-inflammatory cytokine that suppresses host immunity. The promising cytokines, IFN-γ, TNF-α and IL-10, are among the most well-studied cytokines for distinguishing between LTBI and active TB. However, the reported diagnostic value of each cytokine varies depending on the study (13, 51), partly due to differences in the TB antigens used for stimulation. Although the HBHA-specific IL-5, ESAT-6/CFP-10-specific IL-9, and Rv3879c-specific IL-9 levels were notably suppressed in the community exposure group, these cytokines did not effectively distinguish between active PTB patients and household contacts in this study. Few prior reports have explored the utility of these cytokines in differentiating between active PTB and LTBI (13, 52, 53).

This study also demonstrates that MDP-1 and Acr are advantageous TB antigens for eliciting the diagnostic value of these cytokines. MDP-1 and Acr are known to be expressed during latent infection, which is believed to be beneficial for the survival of MTB (16, 19, 20, 54). A limited number of studies have explored MDP-1-specific cytokine responses (35). This is the first study to systematically evaluate the MDP-1-specific cytokine responses with WBS and demonstrate the utility of these cytokines. Among all cytokine-stimulating antigen combinations assessed in the current study, only MDP-1-specific IFN-γ could detect all differences between the active PTB and household contact groups, between the active PTB and community exposure groups, and between the household contact and community exposure groups, as illustrated in **Figure 2** and **Supplementary Figure 3**. This finding suggests that MDP-1-specific IFN-γ can be used as a biomarker for distinguishing among active TB, recent LTBI and remote LTBI. MDP-1 is reportedly responsible for the induction of growth arrest in mycobacteria (21–23), and the expression of MDP-1 was shown to increase during the stationary and dormant phases of *M. smegmatis* (54). Another study demonstrated the pivotal role of MDP-1 in persistent mycobacterial infection, which affected factors such as adaptation to acidic conditions and the macrophage fusion rate (55). In this study, there was a trend toward increased levels of IFN-γ induced by MDP-1 in the community exposure group, followed by the household contact group and then the active PTB group. In groups experiencing persistent infection, where MTB is more stationary or dormant, a stronger immune response against MDP-1 might be detected. Additionally, because the IFN-γ response to MDP-1 is associated with protection against MTB, a strong immune response to MDP-1, as shown in the community exposure group, may have suppressed progression to active TB (56, 57). Regarding Acr, a study from Ethiopia assessed Acr-specific cytokine responses and reported significantly higher levels of TNF-α and IL-10 induced by Acr in the untreated pulmonary TB patient group than in the household contact group using the WBS assay; conversely, IFN-γ showed no significant difference between the two groups, which was consistent with our study findings (58). A strong immune response to Acr has been reported in patients with active TB (32), which may explain the observed immune response to Acr in patients with active TB in the present study and the Ethiopian study.

A stronger IL-10 response was induced in active PTB patients than in household contacts and community exposure subjects when stimulated by Acr. In our previous study using intracellular cytokine staining (ICS), we found that the IL-10 response was more pronounced in patients with active TB and in contacts than in healthy controls (35). It is hypothesized that IL-10 might primarily function to restrict the elimination of pathogens in the initial immune response to MTB, mainly through its inhibitory impacts on the activation of macrophages and the function of DCs (59–61). Previous studies in humans and mice have shown that IL-10 responses and genetic polymorphisms are associated with the development of tuberculosis (62). These findings suggest that MTB might employ a strategy that exploits IL-10 to create a favorable environment by inhibiting the host immune response against the pathogen, enabling MTB to survive and thrive. Based on our findings, we speculate that the immunosuppressive mechanism employed by MTB might be visualized by antigen stimulation using Acr.

A previous study conducted by our group using ICS revealed unique Th1- and non-Th1-type cytokine profiles of CD4+ responses when stimulated with latency-associated MTB antigens (35). However, ICS evaluation is unsuitable for clinical evaluation and screening because it requires special equipment and expertise. This study highlighted the feasibility of WBS for rapid evaluation in clinical practice. Additionally, there are several other advantages of WBS. Compared with peripheral blood mononuclear cell (PBMC) stimulation, WBS allows the assessment of cytokines using a smaller volume of blood (63). WBS also provides a more physiological environment that includes interactions between cells or cells and other components of the blood, such as interfering proteins that occur *in vivo* (64). For example, cytokine production involves cells other than those found among PBMCs, such as platelets and granulocytes (65, 66). Previous studies using cytokine assays have reported discrepant results between WBS and PBMC stimulation (64, 67, 68); thus, proper inferences regarding cytokine trends *in vivo* require evaluation in a physiological environment. In this regard, WBS offers advantages in terms of its feasibility in clinical practice, small specimen requirement and ability to be evaluated under physiological conditions.

A wide variety of specific cytokine responses induced by different MTB antigens have been explored for the differentiation of LTBI and active TB. Furthermore, various efforts have also been made to improve the accuracy of these methods, for example, by combining multiple cytokine responses while using IGRA kits or by using new devices that facilitate standardized immune assays (69, 70). However, there has been a wide range of variations in the results of previous studies (13, 71), and a consensus has not yet been reached regarding the optimal method. These mixed results are partly due to differences in methodology, including the stimulation method used (WBS or stimulation of PBMCs), concentrations of antigens used, availability of costimulation, stimulation duration, TB endemicity at study sites and characteristics of control groups, necessity of specimen transport, and cytokine assessment methods (ELISA or ICS). For a fair comparison, it is desirable to establish a standard methodology for assessing the cytokine responses induced by MTB antigens.

This study has several limitations. This investigation was a single-center study, and the number of participants was small, potentially constraining the statistical robustness and generalizability of the findings, particularly when extrapolating to populations with varying TB burdens or genetic backgrounds. This study also lacked longitudinal data, resulting in the inability to evaluate the temporal changes in the cytokine profiles and their association with subsequent TB progression. Due to the limited application of the NAA test at the study site, the study included active PTB patients for whom molecular biological confirmation was lacking. IGRAs and tuberculin skin tests were not available for the diagnosis of LTBI, and household contacts and community exposure subjects were established as alternative groups for LTBI, as defined by clinical speculation. This limitation introduces uncertainty about the presence of LTBI and may limit the interpretability of the results. Furthermore, it was difficult to compile a TB-uninfected group as a control in the Philippines due to its high TB burden. Although we attempted to control for potential measured confounders, some, such as undiagnosed complications, may still have existed due to the limited diagnostic techniques available at the study site. Our study revealed a sex imbalance in the active PTB group. Although we adjusted for potential confounders, including sex, the likelihood that this imbalance impacted the results was not entirely ruled out, and further confirmation with a more balanced distribution of male and female participants is desirable. Although various types of cytokines have been assessed in previous reports, sample volume limitations restricted the assessment to a small fraction in this study. The inability of the incubator to control the concentration of carbon dioxide and the long-distance transport of specimens may also have affected the concentrations of cytokines. Further data validation is required to confirm our findings.

## 5 Conclusion

This study demonstrated the potential of cytokine profiles induced by latency-associated MTB antigens to discriminate LTBI from active PTB using WBS in countries with a high TB burden. This study identified effective cytokine-MTB antigen combinations, particularly MDP-1-specific IFN-γ, Acr-specific TNF-α and Acr-specific IL-10, and provides the first demonstration of the efficacy of MDP-1-specific cytokine responses for WBS. These promising antigen-specific cytokines are expected to contribute to the detection of LTBI and the prediction of TB progression, which are key to TB control. Further longitudinal studies will provide insights into the relationship between dynamic changes in cytokine profiles and the incidence of TB progression.

## Data availability statement

The raw data supporting the conclusions of this article will be made available by the authors, without undue reservation.

## Ethics statement

The studies involving humans were approved by the Research Ethical and Review Unit of San Lazaro Hospital, the Philippines/School of Tropical Medicine and Global Health, Nagasaki University, Japan. The studies were conducted in accordance with the local legislation and institutional requirements. The participants provided their written informed consent to participate in this study.

## Author contributions

IY: Conceptualization, Data curation, Formal analysis, Investigation, Methodology, Project administration, Resources, Software, Visualization, Writing – original draft, Funding acquisition. NS: Data curation, Investigation, Writing – review & editing. AS: Data curation, Investigation, Writing – review & editing. SS: Data curation, Investigation, Writing – review & editing. AY: Methodology, Resources, Writing – review & editing. YO: Methodology, Resources, Writing – review & editing. HK: Methodology, Resources, Writing – review & editing. AN: Methodology, Resources, Writing – review & editing. SM: Formal analysis, Funding acquisition, Methodology, Resources, Writing – review & editing. SC: Funding acquisition, Methodology, Supervision, Writing – review & editing. TT: Conceptualization, Methodology, Supervision, Validation, Writing – review & editing. YY: Conceptualization, Formal analysis, Funding acquisition, Methodology, Supervision, Validation, Writing – review & editing.

## References

[1] World Health Organization. Latent TB Infection: Updated and consolidated guidelines for programmatic management . Available online at: https://www.who.int/publications/i/item/9789241550239 (Accessed 10 October 2023). 30277688

[2] Pai M Behr MA Dowdy D Dheda K Divangahi M Boehme CC. Tuberculosis. Nat Rev Dis Primers. (2016) 2:16076. doi: 10.1038/nrdp.2016.76 27784885

[3] Chandra P Grigsby SJ Philips JA. Immune evasion and provocation by Mycobacterium tuberculosis. Nat Rev Microbiol. (2022) 20:750–66. doi: 10.1038/s41579-022-00763-4 PMC9310001 35879556

[4] Shah M Dorman SE. Latent tuberculosis infection. N Engl J Med. (2021) 385:2271–80. doi: 10.1056/NEJMcp2108501 34879450

[5] Achkar JM Lawn SD Moosa MY Wright CA Kasprowicz VO. Adjunctive tests for diagnosis of tuberculosis: serology, ELISPOT for site-specific lymphocytes, urinary lipoarabinomannan, string test, and fine needle aspiration. J Infect Dis. (2011) 204 Suppl 4:S1130–1141. doi: 10.1093/infdis/jir450 PMC3192548 21996695

[6] Lawn SD Mwaba P Bates M Piatek A Alexander H Marais BJ. Advances in tuberculosis diagnostics: the Xpert MTB/RIF assay and future prospects for a point-of-care test. Lancet Infect Dis. (2013) 13:349–61. doi: 10.1016/S1473-3099(13)70008-2 PMC4844338 23531388

[7] Boehme CC Nabeta P Henostroza G Raqib R Rahim Z Gerhardt M. Operational feasibility of using loop-mediated isothermal amplification for diagnosis of pulmonary tuberculosis in microscopy centers of developing countries. J Clin Microbiol. (2007) 45:1936–40. doi: 10.1128/JCM.02352-06 PMC1933042 17392443

[8] Metcalfe JZ Everett CK Steingart KR Cattamanchi A Huang L Hopewell PC. Interferon-gamma release assays for active pulmonary tuberculosis diagnosis in adults in low- and middle-income countries: systematic review and meta-analysis. J Infect Dis. (2011) 204 Suppl 4:S1120–1129. doi: 10.1093/infdis/jir410 PMC3192542 21996694

[9] Pai M Menzies D. Interferon-gamma release assays: what is their role in the diagnosis of active tuberculosis? Clin Infect Dis. (2007) 44:74–7. doi: 10.1086/509927 17143819

[10] Rangaka MX Wilkinson KA Glynn JR Ling D Menzies D Mwansa-Kambafwile J. Predictive value of interferon-gamma release assays for incident active tuberculosis: a systematic review and meta-analysis. Lancet Infect Dis. (2012) 12:45–55. doi: 10.1016/S1473-3099(11)70210-9 21846592 PMC3568693

[11] Gong W Wu X. Differential diagnosis of latent tuberculosis infection and active tuberculosis: A key to a successful tuberculosis control strategy. Front Microbiol. (2021) 12:745592. doi: 10.3389/fmicb.2021.745592 34745048 PMC8570039

[12] Pantaleo G Harari A. Functional signatures in antiviral T-cell immunity for monitoring virus-associated diseases. Nat Rev Immunol. (2006) 6:417–23. doi: 10.1038/nri1840 16622477

[13] Meier NR Jacobsen M Ottenhoff THM Ritz N. A systematic review on novel mycobacterium tuberculosis antigens and their discriminatory potential for the diagnosis of latent and active tuberculosis. Front Immunol. (2018) 9:2476. doi: 10.3389/fimmu.2018.02476 30473692 PMC6237970

[14] Mahairas GG Sabo PJ Hickey MJ Singh DC Stover CK. Molecular analysis of genetic differences between Mycobacterium bovis BCG and virulent M. bovis. J Bacteriol. (1996) 178:1274–82. doi: 10.1128/jb.178.5.1274-1282.1996 PMC177799 8631702

[15] van der Wel N Hava D Houben D Fluitsma D van Zon M Pierson J. M. tuberculosis and M. leprae translocate from the phagolysosome to the cytosol in myeloid cells. Cell. (2007) 129:1287–98. doi: 10.1016/j.cell.2007.05.059 17604718

[16] Matsumoto S Yukitake H Furugen M Matsuo T Mineta T Yamada T. Identification of a novel DNA-binding protein from Mycobacterium bovis bacillus Calmette-Guerin. Microbiol Immunol. (1999) 43:1027–36. doi: 10.1111/j.1348-0421.1999.tb01232.x 10609612

[17] Menozzi FD Rouse JH Alavi M Laude-Sharp M Muller J Bischoff R. Identification of a heparin-binding hemagglutinin present in mycobacteria. J Exp Med. (1996) 184:993–1001. doi: 10.1084/jem.184.3.993 9064359 PMC2192777

[18] Sherman DR Voskuil M Schnappinger D Liao R Harrell MI Schoolnik GK. Regulation of the Mycobacterium tuberculosis hypoxic response gene encoding alpha -crystallin. Proc Natl Acad Sci U.S.A. (2001) 98:7534–9. doi: 10.1073/pnas.121172498 PMC34703 11416222

[19] Yuan Y Crane DD Barry CE 3rd. Stationary phase-associated protein expression in Mycobacterium tuberculosis: function of the mycobacterial alpha-crystallin homolog. J Bacteriol. (1996) 178:4484–92. doi: 10.1128/jb.178.15.4484-4492.1996 PMC178214 8755875

[20] Yuan Y Crane DD Simpson RM Zhu YQ Hickey MJ Sherman DR. The 16-kDa alpha-crystallin (Acr) protein of Mycobacterium tuberculosis is required for growth in macrophages. Proc Natl Acad Sci U.S.A. (1998) 95:9578–83. doi: 10.1073/pnas.95.16.9578 PMC21381 9689123

[21] Katsube T Matsumoto S Takatsuka M Okuyama M Ozeki Y Naito M. Control of cell wall assembly by a histone-like protein in Mycobacteria. J Bacteriol. (2007) 189:8241–9. doi: 10.1128/JB.00550-07 PMC2168677 17873049

[22] Matsumoto S Furugen M Yukitake H Yamada T. The gene encoding mycobacterial DNA-binding protein I (MDPI) transformed rapidly growing bacteria to slowly growing bacteria. FEMS Microbiol Lett. (2000) 182:297–301. doi: 10.1111/j.1574-6968.2000.tb08911.x 10620682

[23] Savitskaya A Nishiyama A Yamaguchi T Tateishi Y Ozeki Y Nameta M. C-terminal intrinsically disordered region-dependent organization of the mycobacterial genome by a histone-like protein. Sci Rep. (2018) 8:8197. doi: 10.1038/s41598-018-26463-9 29844400 PMC5974015

[24] Nishiyama A Shimizu M Narita T Kodera N Ozeki Y Yokoyama A. Dynamic action of an intrinsically disordered protein in DNA compaction that induces mycobacterial dormancy. Nucleic Acids Res. (2023) 52(2):816–30. doi: 10.1093/nar/gkad1149 PMC10810275 38048321

[25] Niki M Niki M Tateishi Y Ozeki Y Kirikae T Lewin A. A novel mechanism of growth phase-dependent tolerance to isoniazid in mycobacteria. J Biol Chem. (2012) 287:27743–52. doi: 10.1074/jbc.M111.333385 PMC3431685 22648414

[26] Sakatos A Babunovic GH Chase MR Dills A Leszyk J Rosebrock T. Posttranslational modification of a histone-like protein regulates phenotypic resistance to isoniazid in mycobacteria. Sci Adv. (2018) 4:eaao1478. doi: 10.1126/sciadv.aao1478 29732401 PMC5931751

[27] Wayne LG Hayes LG. An in *vitro* model for sequential study of shiftdown of Mycobacterium tuberculosis through two stages of nonreplicating persistence. Infect Immun. (1996) 64:2062–9. doi: 10.1128/iai.64.6.2062-2069.1996 PMC174037 8675308

[28] Pethe K Bifani P Drobecq H Sergheraert C Debrie AS Locht C. Mycobacterial heparin-binding hemagglutinin and laminin-binding protein share antigenic methyllysines that confer resistance to proteolysis. Proc Natl Acad Sci USA. (2002) 99:10759–64. doi: 10.1073/pnas.162246899 PMC125037 12149464

[29] Aoki K Matsumoto S Hirayama Y Wada T Ozeki Y Niki M. Extracellular mycobacterial DNA-binding protein 1 participates in mycobacterium-lung epithelial cell interaction through hyaluronic acid. J Biol Chem. (2004) 279:39798–806. doi: 10.1074/jbc.M402677200 15234978

[30] Niki M Yoshiyama T Miyamoto Y Okumura M Niki M Oinuma KI. Longitudinal evaluation of humoral immunity and bacterial and clinical parameters reveals that antigen-specific antibodies suppress inflammatory responses in active tuberculosis patients. J Immunol Res. (2018) 2018:4928757. doi: 10.1155/2018/4928757 30069487 PMC6057312

[31] Ohara Y Ozeki Y Tateishi Y Mashima T Arisaka F Tsunaka Y. Significance of a histone-like protein with its native structure for the diagnosis of asymptomatic tuberculosis. PloS One. (2018) 13:e0204160. doi: 10.1371/journal.pone.0204160 30359374 PMC6201868

[32] Osada-Oka M Tateishi Y Hirayama Y Ozeki Y Niki M Kitada S. Antigen 85A and mycobacterial DNA-binding protein 1 are targets of immunoglobulin G in individuals with past tuberculosis. Microbiol Immunol. (2013) 57:30–7. doi: 10.1111/j.1348-0421.2012.12005.x 23157580

[33] Tang J Huang Y Cai Z Ma Y. Mycobacterial heparin-binding hemagglutinin (HBHA)-induced interferon-gamma release assay (IGRA) for discrimination of latent and active tuberculosis: A systematic review and meta-analysis. PloS One. (2021) 16:e0254571. doi: 10.1371/journal.pone.0254571 34270559 PMC8284824

[34] Yamashita Y Hoshino Y Oka M Matsumoto S Ariga H Nagai H. Multicolor flow cytometric analyses of CD4+ T cell responses to Mycobacterium tuberculosis-related latent antigens. Jpn J Infect Dis. (2013) 66:207–15. doi: 10.7883/yoken.66.207 23698481

[35] Yamashita Y Oe T Kawakami K Osada-Oka M Ozeki Y Terahara K. CD4(+) T responses other than th1 type are preferentially induced by latency-associated antigens in the state of latent mycobacterium tuberculosis infection. Front Immunol. (2019) 10:2807. doi: 10.3389/fimmu.2019.02807 31849981 PMC6897369

[36] World Health Organization. Global Tuberculosis Report 2022 . Available online at: https://www.who.int/teams/global-tuberculosis-programme/tb-reports/global-tuberculosis-report-2022 (Accessed 10 October 2023).

[37] Ding C Hu M Guo W Hu W Li X Wang S. Prevalence trends of latent tuberculosis infection at the global, regional, and country levels from 1990-2019. Int J Infect Dis. (2022) 122:46–62. doi: 10.1016/j.ijid.2022.05.029 35577247

[38] Cox SE Edwards T Faguer BN Ferrer JP Suzuki SJ Koh M. Patterns of non-communicable comorbidities at start of tuberculosis treatment in three regions of the Philippines: The St-ATT cohort. PloS Glob Public Health. (2021) 1:e0000011. doi: 10.1371/journal.pgph.0000011 36962076 PMC10021424

[39] Maekura R Kitada S Osada-Oka M Tateishi Y Ozeki Y Fujicawa T. Serum antibody profiles in individuals with latent Mycobacterium tuberculosis infection. Microbiol Immunol. (2019) 63:130–8. doi: 10.1111/1348-0421.12674 PMC6767593 30851131

[40] Ishikawa S Ozeki Y Suga S Mukai Y Kobayashi H Inouchi E. Monitoring IgG against Mycobacterium tuberculosis proteins in an Asian elephant cured of tuberculosis that developed from long-term latency. Sci Rep. (2022) 12:4310. doi: 10.1038/s41598-022-08228-7 35279668 PMC8917326

[41] Saravia J Chapman NM Chi H. Helper T cell differentiation. Cell Mol Immunol. (2019) 16:634–43. doi: 10.1038/s41423-019-0220-6 PMC6804569 30867582

[42] Kimura D Miyakoda M Kimura K Honma K Hara H Yoshida H. Interleukin-27-producing CD4(+) T cells regulate protective immunity during malaria parasite infection. Immunity. (2016) 44:672–82. doi: 10.1016/j.immuni.2016.02.011 26968425

[43] Behr MA Wilson MA Gill WP Salamon H Schoolnik GK Rane S. Comparative genomics of BCG vaccines by whole-genome DNA microarray. Science. (1999) 284:1520–3. doi: 10.1126/science.284.5419.1520 10348738

[44] Behr MA Edelstein PH Ramakrishnan L. Revisiting the timetable of tuberculosis. BMJ. (2018) 362:k2738. doi: 10.1136/bmj.k2738 30139910 PMC6105930

[45] Wiker HG Mustafa T Bjune GA Harboe M. Evidence for waning of latency in a cohort study of tuberculosis. BMC Infect Dis. (2010) 10:37. doi: 10.1186/1471-2334-10-37 20178619 PMC2843612

[46] Getahun H Matteelli A Abubakar I Aziz MA Baddeley A Barreira D. Management of latent Mycobacterium tuberculosis infection: WHO guidelines for low tuberculosis burden countries. Eur Respir J. (2015) 46:1563–76. doi: 10.1183/13993003.01245-2015 PMC4664608 26405286

[47] Kahwati LC Feltner C Halpern M Woodell CL Boland E Amick HR. Primary care screening and treatment for latent tuberculosis infection in adults: evidence report and systematic review for the US preventive services task force. JAMA. (2016) 316:970–83. doi: 10.1001/jama.2016.10357 27599332

[48] Froberg G Wahren Borgstrom E Chryssanthou E Correia-Neves M Kallenius G Bruchfeld J. A new mathematical model to identify contacts with recent and remote latent tuberculosis. ERJ Open Res. (2019) 5. doi: 10.1183/23120541.00078-2019 PMC6556559 31205929

[49] Global Burden of Disease Collaborative Network. Available online at: http://ghdx.healthdata.org/gbd-results-tool (Accessed 20 February 2024).

[50] Paton NI Borand L Benedicto J Kyi MM Mahmud AM Norazmi MN. Diagnosis and management of latent tuberculosis infection in Asia: Review of current status and challenges. Int J Infect Dis. (2019) 87:21–9. doi: 10.1016/j.ijid.2019.07.004 31301458

[51] Wei Z Li Y Wei C Li Y Xu H Wu Y. The meta-analysis for ideal cytokines to distinguish the latent and active TB infection. BMC Pulm Med. (2020) 20:248. doi: 10.1186/s12890-020-01280-x 32948170 PMC7502022

[52] Hussain R Kaleem A Shahid F Dojki M Jamil B Mehmood H. Cytokine profiles using whole-blood assays can discriminate between tuberculosis patients and healthy endemic controls in a BCG-vaccinated population. J Immunol Methods. (2002) 264:95–108. doi: 10.1016/s0022-1759(02)00092-3 12191514

[53] Wu B Huang C Kato-Maeda M Hopewell PC Daley CL Krensky AM. IL-9 is associated with an impaired Th1 immune response in patients with tuberculosis. Clin Immunol. (2008) 126:202–10. doi: 10.1016/j.clim.2007.09.009 18032114

[54] Enany S Yoshida Y Tateishi Y Ozeki Y Nishiyama A Savitskaya A. Mycobacterial DNA-binding protein 1 is critical for long term survival of Mycobacterium smegmatis and simultaneously coordinates cellular functions. Sci Rep. (2017) 7:6810. doi: 10.1038/s41598-017-06480-w 28754952 PMC5533761

[55] Kunisch R Kamal E Lewin A. The role of the mycobacterial DNA-binding protein 1 (MDP1) from Mycobacterium bovis BCG in host cell interaction. BMC Microbiol. (2012) 12:165. doi: 10.1186/1471-2180-12-165 22863261 PMC3438132

[56] Matsumoto S Matsumoto M Umemori K Ozeki Y Furugen M Tatsuo T. DNA augments antigenicity of mycobacterial DNA-binding protein 1 and confers protection against Mycobacterium tuberculosis infection in mice. J Immunol. (2005) 175:441–9. doi: 10.4049/jimmunol.175.1.441 15972678

[57] Maeyama JI Iho S Suzuki F Hayashi D Yamamoto T Yamazaki T. Evaluation of a booster tuberculosis vaccine containing mycobacterial DNA-binding protein 1 and CpG oligodeoxynucleotide G9.1 using a Guinea pig model that elicits immunity to Bacillus Calmette-Guerin. Tuberculosis (Edinb). (2021) 128:102067. doi: 10.1016/j.tube.2021.102067 33752142

[58] Belay M Legesse M Mihret A Bekele Y Ottenhoff TH Franken KL. Pro- and anti-inflammatory cytokines against Rv2031 are elevated during latent tuberculosis: a study in cohorts of tuberculosis patients, household contacts and community controls in an endemic setting. PloS One. (2015) 10:e0124134. doi: 10.1371/journal.pone.0124134 25897840 PMC4405476

[59] Redford PS Murray PJ O’Garra A. The role of IL-10 in immune regulation during M. tuberculosis infection. Mucosal Immunol. (2011) 4:261–70. doi: 10.1038/mi.2011.7 21451501

[60] Cyktor JC Carruthers B Kominsky RA Beamer GL Stromberg P Turner J. IL-10 inhibits mature fibrotic granuloma formation during Mycobacterium tuberculosis infection. J Immunol. (2013) 190:2778–90. doi: 10.4049/jimmunol.1202722 PMC3594073 23396944

[61] Moreira-Teixeira L Redford PS Stavropoulos E Ghilardi N Maynard CL Weaver CT. T cell-derived IL-10 impairs host resistance to mycobacterium tuberculosis infection. J Immunol. (2017) 199:613–23. doi: 10.4049/jimmunol.1601340 PMC5502318 28584007

[62] Cooper AM. Cell-mediated immune responses in tuberculosis. Annu Rev Immunol. (2009) 27:393–422. doi: 10.1146/annurev.immunol.021908.132703 19302046 PMC4298253

[63] Deenadayalan A Maddineni P Raja A. Comparison of whole blood and PBMC assays for T-cell functional analysis. BMC Res Notes. (2013) 6:120. doi: 10.1186/1756-0500-6-120 23531281 PMC3616860

[64] Silva D Ponte CG Hacker MA Antas PR. A whole blood assay as a simple, broad assessment of cytokines and chemokines to evaluate human immune responses to Mycobacterium tuberculosis antigens. Acta Trop. (2013) 127:75–81. doi: 10.1016/j.actatropica.2013.04.002 23571106

[65] Kany S Vollrath JT Relja B. Cytokines in inflammatory disease. Int J Mol Sci. (2019) 20. doi: 10.3390/ijms20236008 PMC6929211 31795299

[66] Aslam R Speck ER Kim M Crow AR Bang KW Nestel FP. Platelet Toll-like receptor expression modulates lipopolysaccharide-induced thrombocytopenia and tumor necrosis factor-alpha production *in vivo* . Blood. (2006) 107:637–41. doi: 10.1182/blood-2005-06-2202 16179373

[67] Loxton AG Black GF Stanley K Walzl G. Heparin-binding hemagglutinin induces IFN-gamma(+) IL-2(+) IL-17(+) multifunctional CD4(+) T cells during latent but not active tuberculosis disease. Clin Vaccine Immunol. (2012) 19:746–51. doi: 10.1128/CVI.00047-12 PMC3346316 22461525

[68] Silberer J Ihorst G Kopp MV. Cytokine levels in supernatants of whole blood and mononuclear cell cultures in adults and neonates reveal significant differences with respect to interleukin-13 and interferon-gamma. Pediatr Allergy Immunol. (2008) 19:140–7. doi: 10.1111/j.1399-3038.2007.00605.x 18257902

[69] Duffy D Nemes E Llibre A Rouilly V Musvosvi M Smith N. Immune profiling enables stratification of patients with active tuberculosis disease or mycobacterium tuberculosis infection. Clin Infect Dis. (2021) 73:e3398–408. doi: 10.1093/cid/ciaa1562 PMC8563210 33059361

[70] Frahm M Goswami ND Owzar K Hecker E Mosher A Cadogan E. Discriminating between latent and active tuberculosis with multiple biomarker responses. Tuberculosis (Edinb). (2011) 91:250–6. doi: 10.1016/j.tube.2011.02.006 PMC3090479 21393062

[71] Carranza C Pedraza-Sanchez S de Oyarzabal-Mendez E Torres M. Diagnosis for latent tuberculosis infection: new alternatives. Front Immunol. (2020) 11:2006. doi: 10.3389/fimmu.2020.02006 33013856 PMC7511583

