# Supplementary material for: Evaluation of cytokine profiles related to Mycobacterium tuberculosis latent antigens using a whole-blood assay in the Philippines
Source: Front Immunol. 2024 Apr 10;15:1330796. doi: 10.3389/fimmu.2024.1330796 (PMC11044679; doi:10.3389/fimmu.2024.1330796)
Supplement: Supplementary file 1 [file DataSheet_1.docx]

**Supplementary Table 1.** Basic characteristics of the compared groups: “active PTB” group versus group of “household contacts and community exposures”.

|  |  | Active PTB (n=15) | HHCs + CEs (n=63) | p value |
| --- | --- | --- | --- | --- |
| Age | year |  |  |  |
|  | median. (range) | 36.0 (19.0–49.0) | 44.0 (18.0–79.0) | 0.044 ‡ |
| Female | no. (%) | 2 (13.3) | 42 (66.7) | <0.001 † |
| BMI | kg/m^2 |  |  |  |
|  | median. (range) | 18.7 (14.1–23.3) | 24.4 (17.0–39.0) | <0.001 ‡ |
| Comorbidity | no. (%) | 0 (0.0) | 3 (4.8) | 1† |
| CBC count | median. (range) |  |  |  |
| WBC | 10^3/µl | 9.1 (6.5–13.4) | 7.5 (3.8–13.3) | 0.016 ‡ |
| Neutrophils | 10^3/µl | 6.3 (2.9–10.8) | 4.1 (2.0–8.6) | <0.001 ‡ |
|  | % | 68.0 (31.8–82.1) | 54.3 (39.1–68.8) | <0.001 ‡ |
| Lymphocytes | 10^3/µl | 1.6 (1.0–3.0) | 2.6 (0.7–4.6) | <0.001 ‡ |
|  | % | 18.5 (8.4–32.7) | 34.7 (19.5–50.7) | <0.001 ‡ |
| Monocytes | 10^3/µl | 0.7 (0.4–1.3) | 0.5 (0.2–0.9) | <0.001 ‡ |
|  | % | 8.2 (4.5–11.2) | 6.8 (4.1–9.3) | 0.007 ‡ |
| Eosinophils | 10^3/µl | 0.43 (0.00–2.76) | 0.30 (0.03–1.11) | 0.727 ‡ |
|  | % | 5.0 (0.0–30.3) | 4.0 (0.4–12.4) | 0.443 ‡ |

The comparisons between two groups were performed using † Fisher’s exact test for categorical variables or ‡ the Mann‒Whitney U test for continuous variables.

**Rv3879c**

AAGCTTGTTCATATGAGCATTACACGGCCAACGGGTAGCTATGCTCGCCAGATGCTCGACCCAGGCGGGTGGGTTGAGGCAGACGAAGATACGTTCTATGACCGCGCTCAGGAGTACAGCCAGGTCCTGCAGCGCGTGACGGACGTTCTGGACACTTGCCGCCAGCAAAAGGGTCACGTGTTTGAAGGCGGCCTGTGGAGTGGTGGAGCGGCTAATGCCGCAAATGGCGCCCTGGGGGCCAACATCAATCAGCTCATGACCCTGCAGGATTACTTAGCGACCGTTATCACTTGGCACCGCCACATTGCGGGCTTAATCGAACAGGCGAAAAGCGACATTGGCAACAACGTCGATGGCGCCCAACGGGAGATTGATATTCTGGAAAATGACCCAAGTCTCGATGCGGATGAACGCCACACCGCGATCAACTCGTTGGTAACGGCGACCCATGGTGCCAACGTTAGCCTGGTGGCCGAAACCGCTGAGCGTGTGCTAGAAAGCAAGAACTGGAAACCACCGAAGAACGCACTGGAGGATCTATTGCAACAAAAATCGCCGCCTCCGCCGGACGTTCCGACCTTGGTGGTCCCGTCACCGGGCACCCCGGGGACGCCGGGCACTCCGATCACGCCCGGAACACCGATTACGCCAGGAACGCCTATTACCCCGATTCCCGGCGCCCCGGTCACTCCCATTACGCCCACTCCGGGGACCCCGGTAACCCCAGTTACACCTGGCAAACCGGTGACCCCTGTCACCCCGGTCAAACCTGGGACCCCCGGCGAACCGACCCCGATTACTCCGGTTACCCCGCCGGTAGCTCCCGCAACCCCCGCTACCCCGGCAACTCCCGTGACCCCAGCGCCTGCACCACATCCGCAACCAGCTCCTGCACCGGCCCCGTCACCAGGCCCACAGCCCGTAACACCAGCCACTCCGGGCCCGAGTGGTCCGGCAACCCCGGGCACGCCGGGAGGCGAACCTGCCCCGCACGTTAAACCTGCCGCGTTAGCCGAACAGCCTGGGGTTCCGGGTCAACATGCAGGTGGCGGAACGCAGAGTGGGCCGGCGCATGCTGATGAGTCTGCCGCGTCTGTCACGCCGGCCGCAGCAAGCGGTGTTCCAGGTGCCCGTGCAGCAGCAGCCGCGCCATCCGGTACTGCGGTGGGTGCTGGCGCCCGTTCATCGGTGGGGACAGCTGCTGCCTCCGGTGCCGGTTCGCATGCGGCGACCGGTCGCGCACCTGTAGCGACATCCGATAAAGCGGCTGCACCGAGTACCCGCGCGGCCTCTGCGCGTACGGCACCACCGGCTCGCCCTCCCTCCACGGATCACATAGACAAACCTGACCGCAGCGAATCTGCGGATGATGGCACCCCAGTAAGCATGATCCCGGTTAGCGCGGCTAGAGCGGCTCGAGATGCGGCCACGGCCGCAGCGTCTGCGCGTCAACGTGGTCGCGGCGATGCGCTGCGACTGGCGCGTCGGATTGCGGCAGCTCTGAATGCGTCGGACAATAACGCCGGTGATTACGGTTTCTTTTGGATCACAGCAGTTACTACCGATGGTTCGATCGTGGTGGCGAATAGCTATGGCCTTGCGTACATCCCAGATGGCATGGAATTACCGAACAAAGTATACCTGGCTAGCGCCGATCATGCGATCCCGGTGGATGAAATAGCCCGTTGTGCGACCTATCCGGTCCTGGCGGTTCAGGCGTGGGCGGCGTTTCATGATATGACACTGCGTGCCGTGATTGGAACCGCAGAACAGCTGGCTTCCTCGGATCCGGGCGTCGCGAAAATTGTGTTGGAACCGGACGACATTCCTGAATCAGGCAAGATGACTGGACGCTCCAGGCTGGAGGTGGTCGATCCTTCAGCTGCAGCACAGCTGGCGGATACCACAGACCAACGCCTGCTGGATCTTCTGCCGCCGGCTCCGGTAGATGTCAATCCCCCGGGTGATGAGCGTCACATGCTTTGGTTCGAACTGATGAAACCGATGACCTCTACCGCGACGGGTCGCGAAGCGGCCCATCTCCGTGCGTTTCGGGCCTATGCCGCGCATAGTCAGGAAATCGCATTACATCAGGCCCATACGGCCACCGATGCAGCAGTGCAACGTGTGGCCGTGGCGGACTGGTTGTATTGGCAGTATGTGACGGGGTTGCTTGACCGTGCCTTAGCCGCGGCATGCCATCACCATCACCACCATTAATAGGTACCGAATTC

**HBHA**

CATATGGCTGAAAACTCGAACATTGATGACATCAAGGCTCCGTTGCTTGCCGCGCTTGGAGCGGCCGACCTGGCCTTGGCCACTGTCAACGAGTTGATCACGAACCTGCGTGAGCGTGCGGAGGAGACTCGTACGGACACCCGCAGCCGGGTCGAGGAGAGCCGTGCTCGCCTGACCAAGCTGCAGGAAGATCTGCCCGAGCAGCTCACCGAGCTGCGTGAGAAGTTCACCGCCGAGGAGCTGCGTAAGGCCGCCGAGGGCTACCTCGAGGCCGCGACTAGCCGGTACAACGAGCTGGTCGAGCGCGGTGAGGCCGCTCTAGAGCGGCTGCGCAGCCAGCAGAGCTTCGAGGAAGTGTCGGCGCGCGCCGAAGGCTACGTGGACCAGGCGGTGGAGTTGACCCAGGAGGCGTTGGGTACGGTCGCATCGCAGACCCGCGCGGTCGGTGAGCGTGCCGCCAAGCTGGTCGGCATCGAGCTGCCTAAGAAGGCTGCTCCGGCCAAGAAGGCCGCTCCGGCCAAGAAGGCCGCTCCGGCCAAGAAGGCGGCGGCCAAGAAGGCGCCCGCGAAGAAGGCGGCGGCCAAGAAGGTCACCCAGAAGCACCACCACCACCACCACTAGGGTACC

**Supplementary Figure 1.** Synthetic genes used for the expression of Rv3879c and HBHA.

Nde1 sites are indicated in red. 6xHIS Tags are written in blue. Stop codons are underlined. C-terminal Kpn1 and HindIII sites are marked by purple.

**
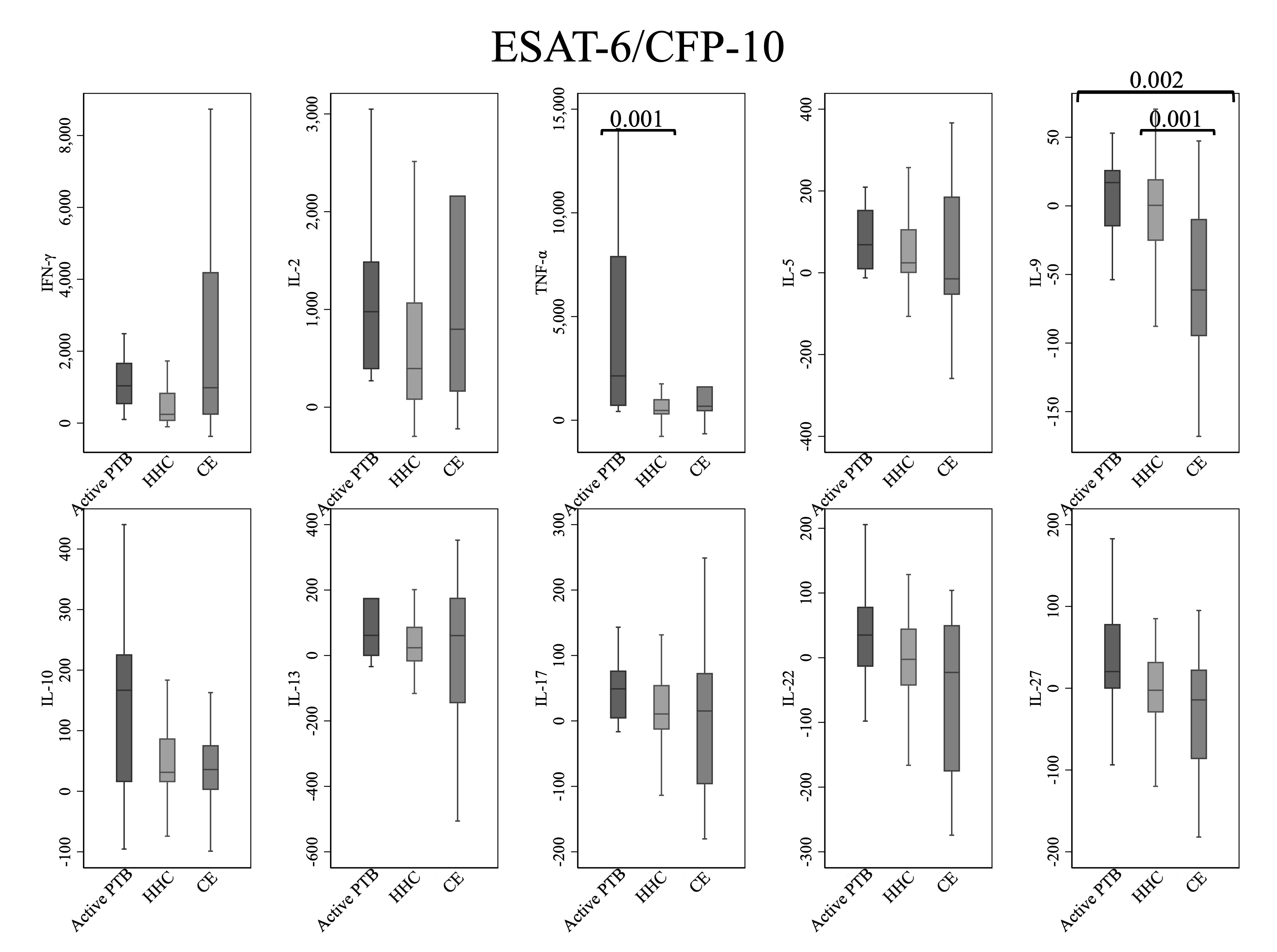
**

**
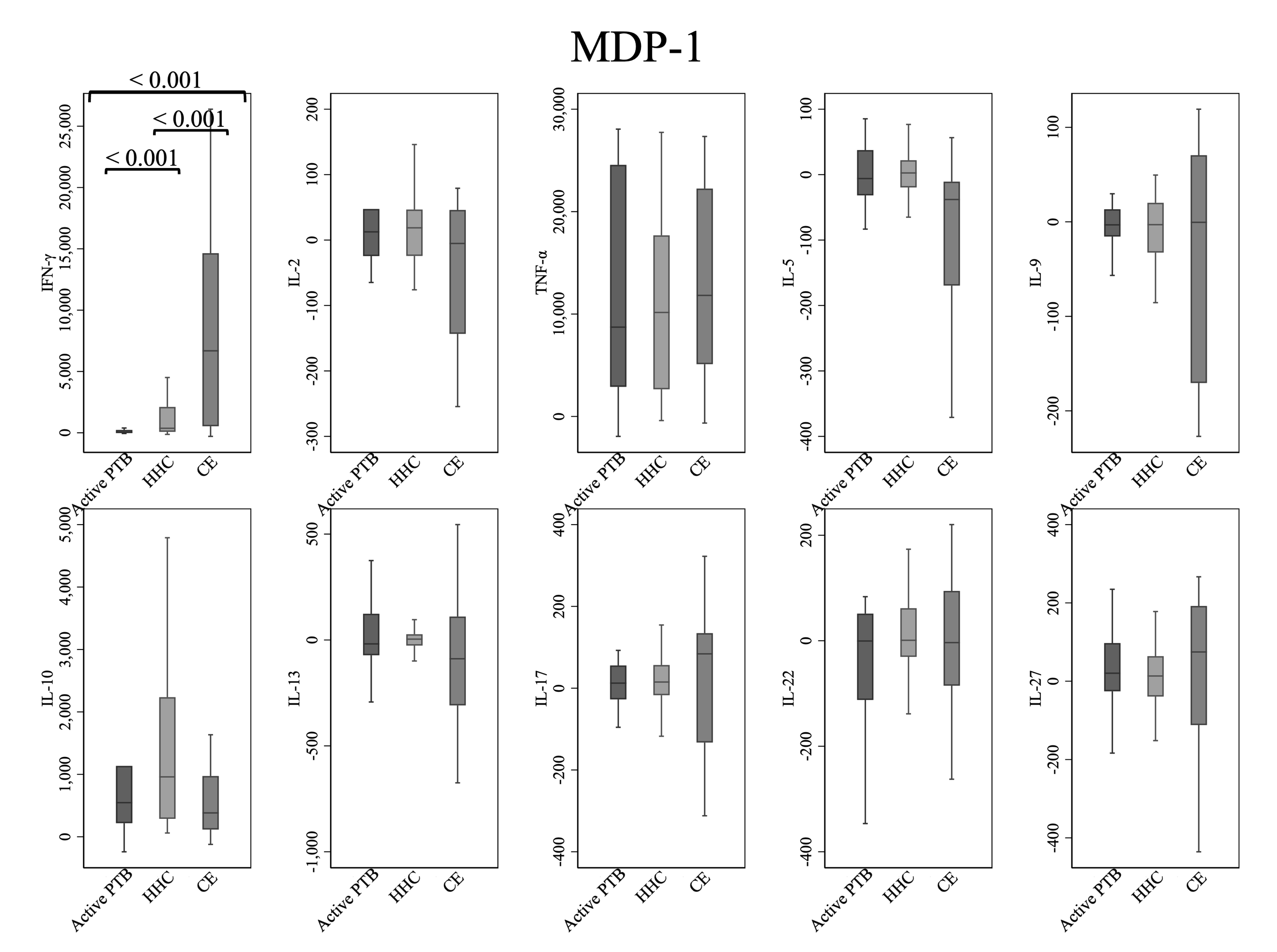
**

**
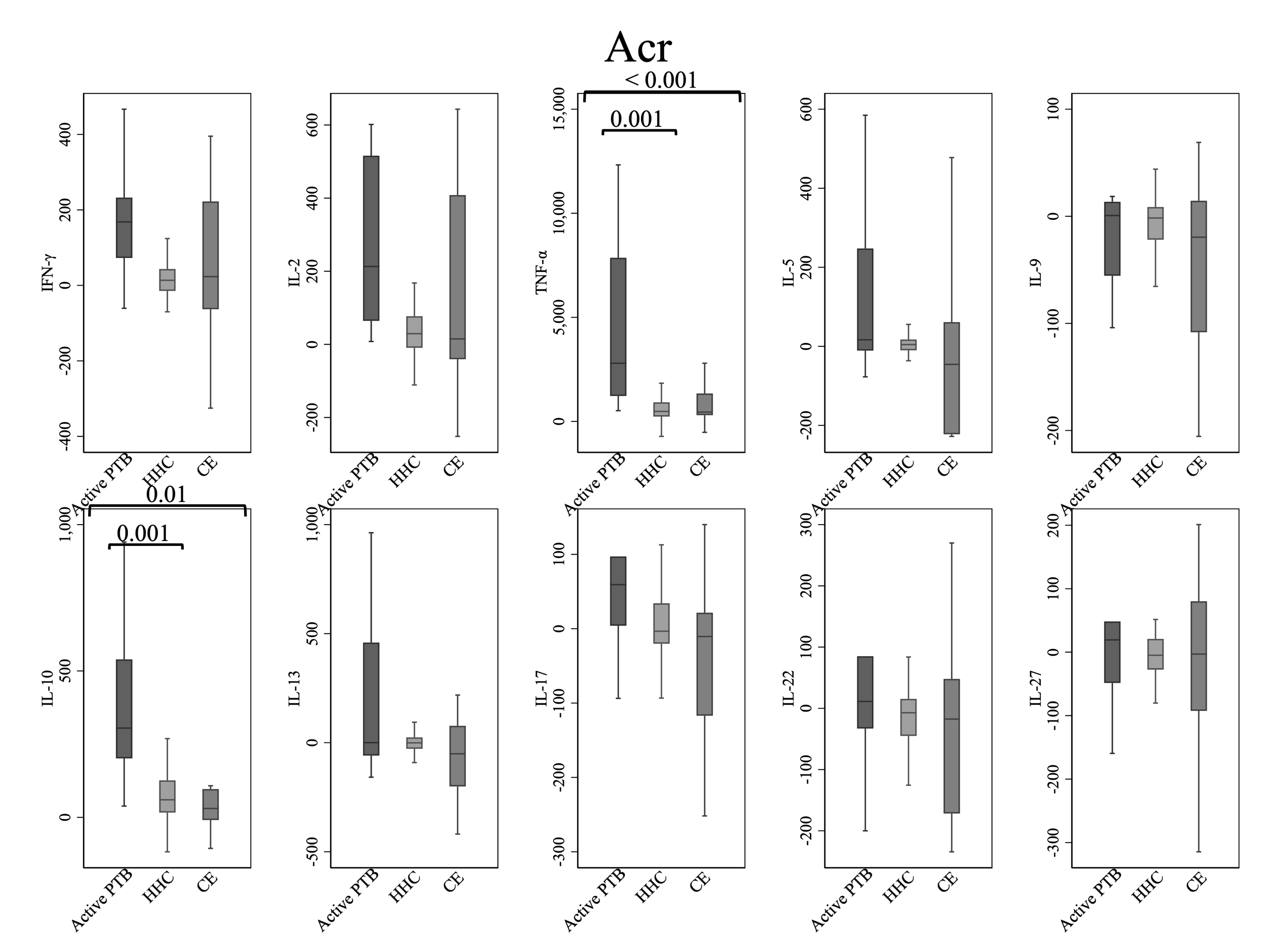
**

**
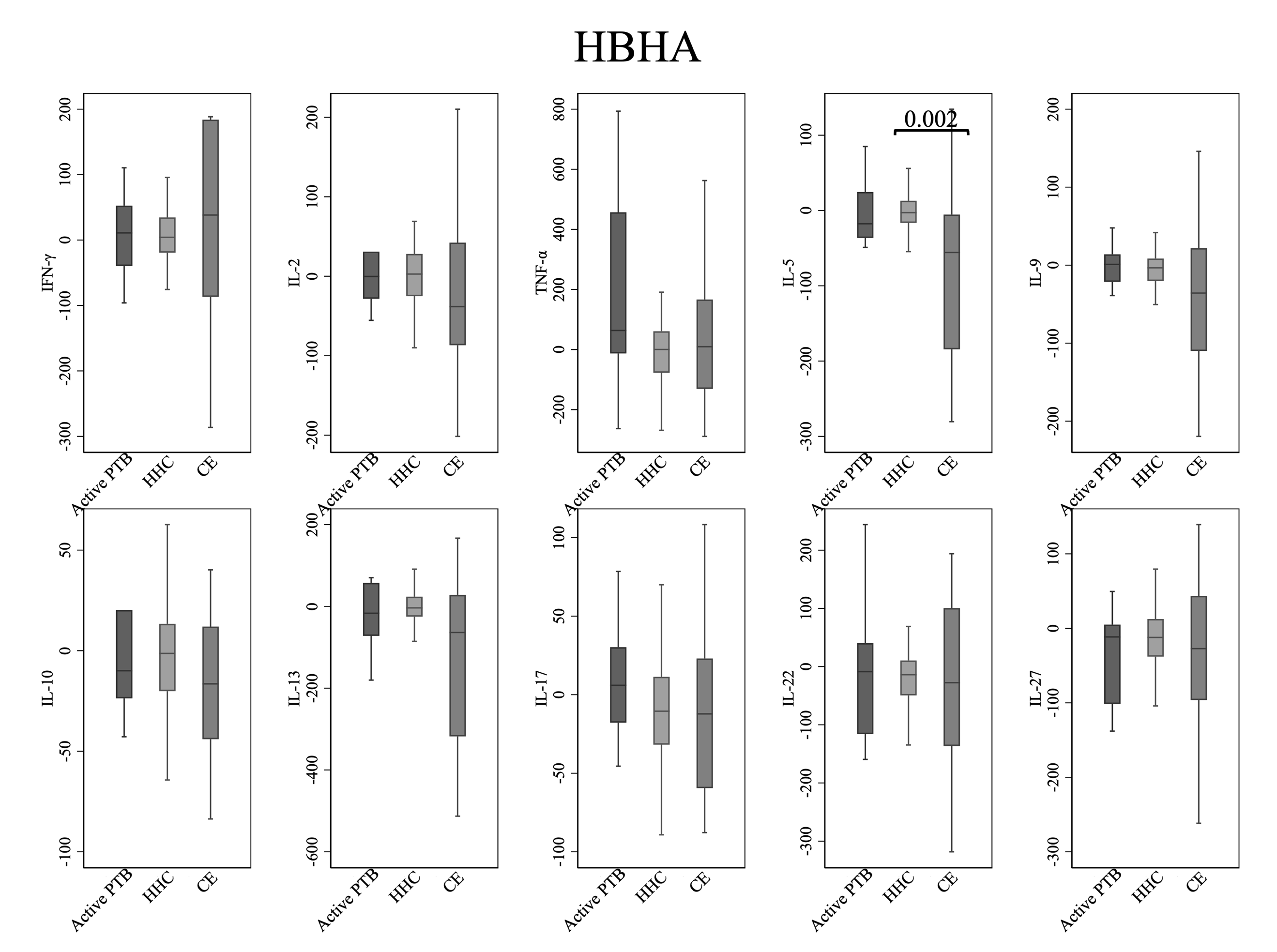
**

**
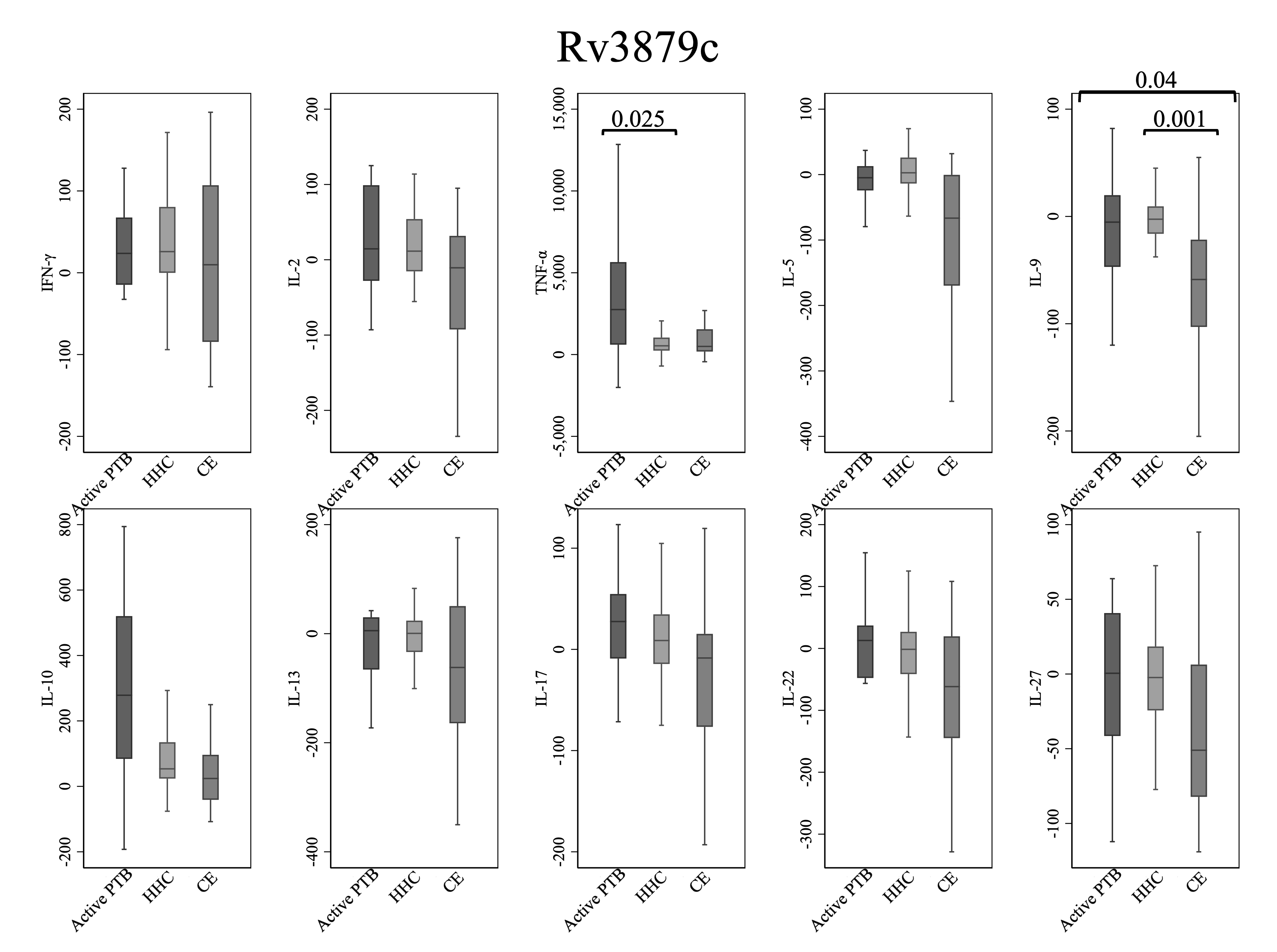
**

**Supplementary Figure 2.** Cytokine responses without outside values for each antigen (box and whisker plots).

The X-axis shows the participant groups. The Y-axis shows the concentrations of cytokines (pg/mL for IFN-γ, IL-2, TNF-α, IL-5, IL-9, IL-10, IL-13 and IL-17; ng/mL for IL-22 and IL-27). The box and whisker plots show the cytokine responses without outside values for each antigen. The boxes show the median and interquartile values, and the whiskers represent the upper and lower adjacent values. The numbers in the figure indicate adjusted p values. Comparisons of the concentrations of each cytokine among active PTB patients, household contacts and community exposures subjects were performed using the Kruskal‒Wallis test followed by Dunn’s *post hoc* test with Holm adjustment. We used linear regression to adjust for the potential confounders age, sex and BMI. Active PTB: active pulmonary tuberculosis, HHC: household contact, CE: community exposure.


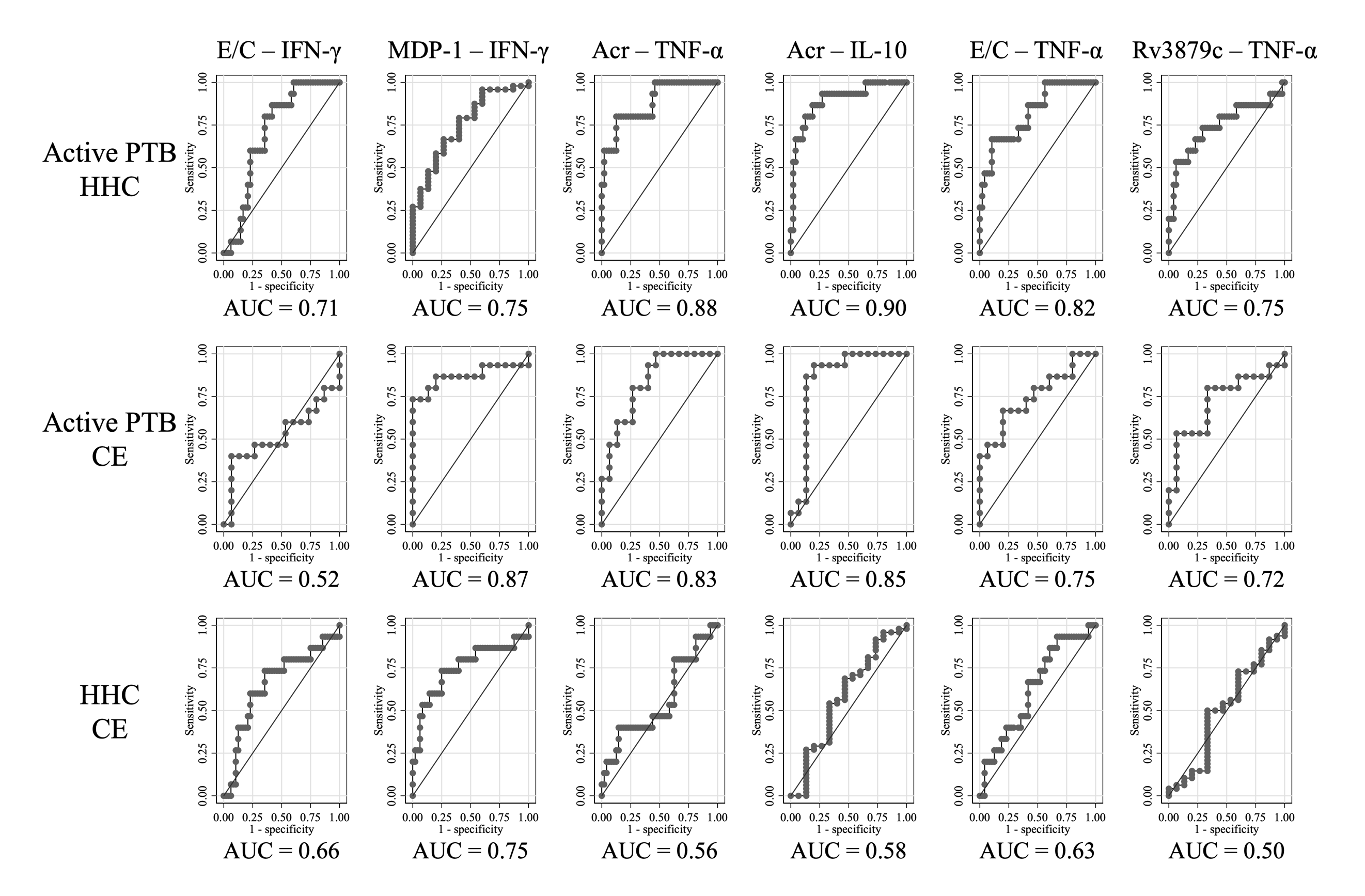


**Supplementary Figure 3.** Receiver operating characteristic curves for the discriminatory performance.

Receiver operating characteristic (ROC) curves depicting the discriminatory performance of each MTB antigen-specific cytokine, including MDP-1-specific IFN-γ, Acr-specific TNF-α, Acr-specific IL-10, ESAT-6/CFP-10-specific TNF-α and Rv3879c-specific TNF-α, were generated. For comparison, the ROC curves for ESAT-6/CFP-10-specific IFN-γ are also shown. These curves illustrate the differentiation between active PTB and household contacts, between active PTB and community exposure subjects, and between household contacts and community exposure subjects.

E/C-IFN-γ: ESAT6/CFP-10-specific IFN-γ, MDP-1-IFN-γ: MDP-1-specific IFN-γ, Acr-TNF-α: Acr-specific TNF-α, Acr-IL-10: Acr-specific IL-10, E/C-TNF-α: ESAT6/CFP-10-specific TNF-α, Rv3879c-TNF-α: Rv3879c-specific TNF-α, Active PTB: active pulmonary tuberculosis, HHC: household contact, CE: community exposure, AUC: area under the ROC curve.


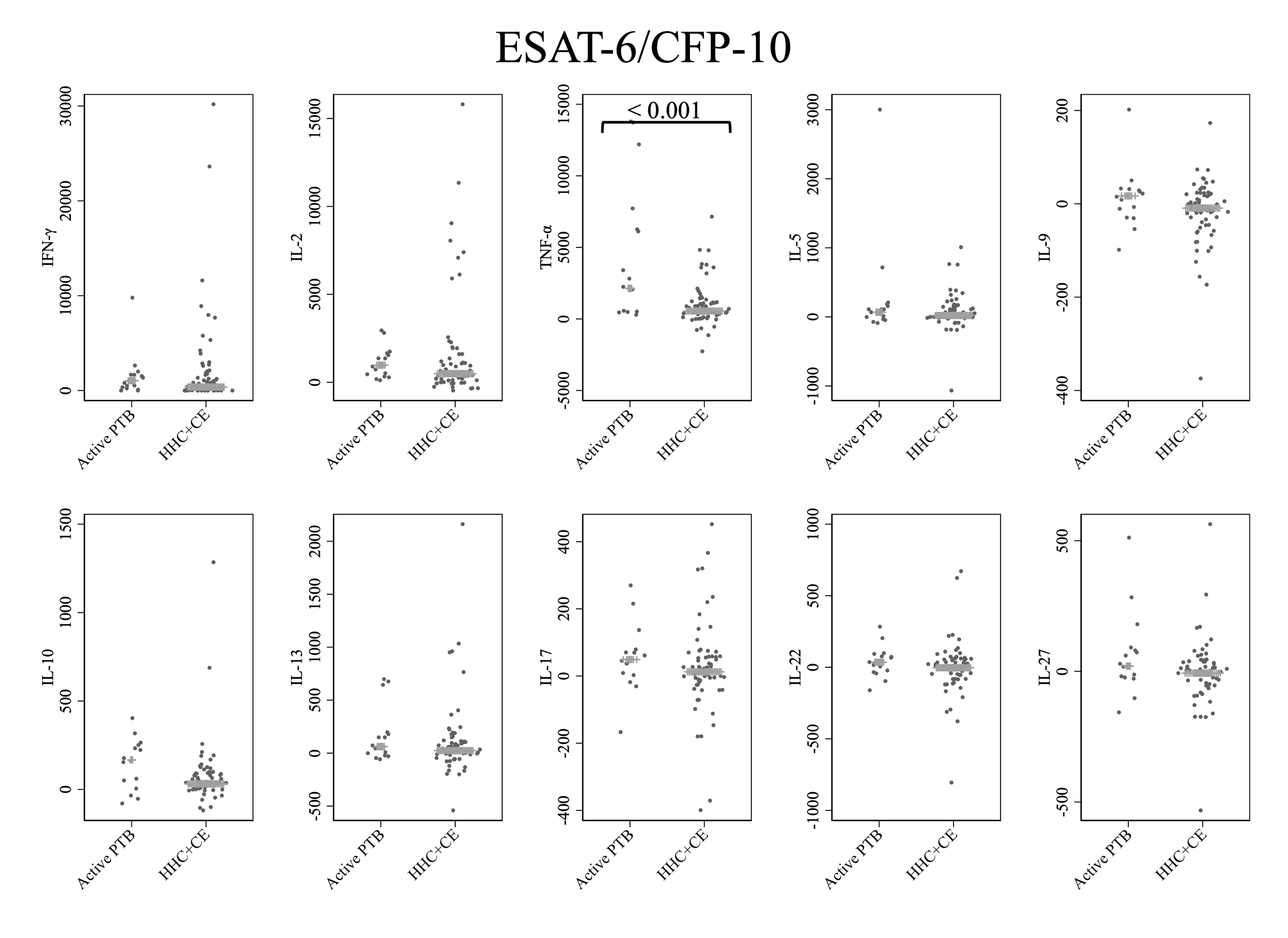


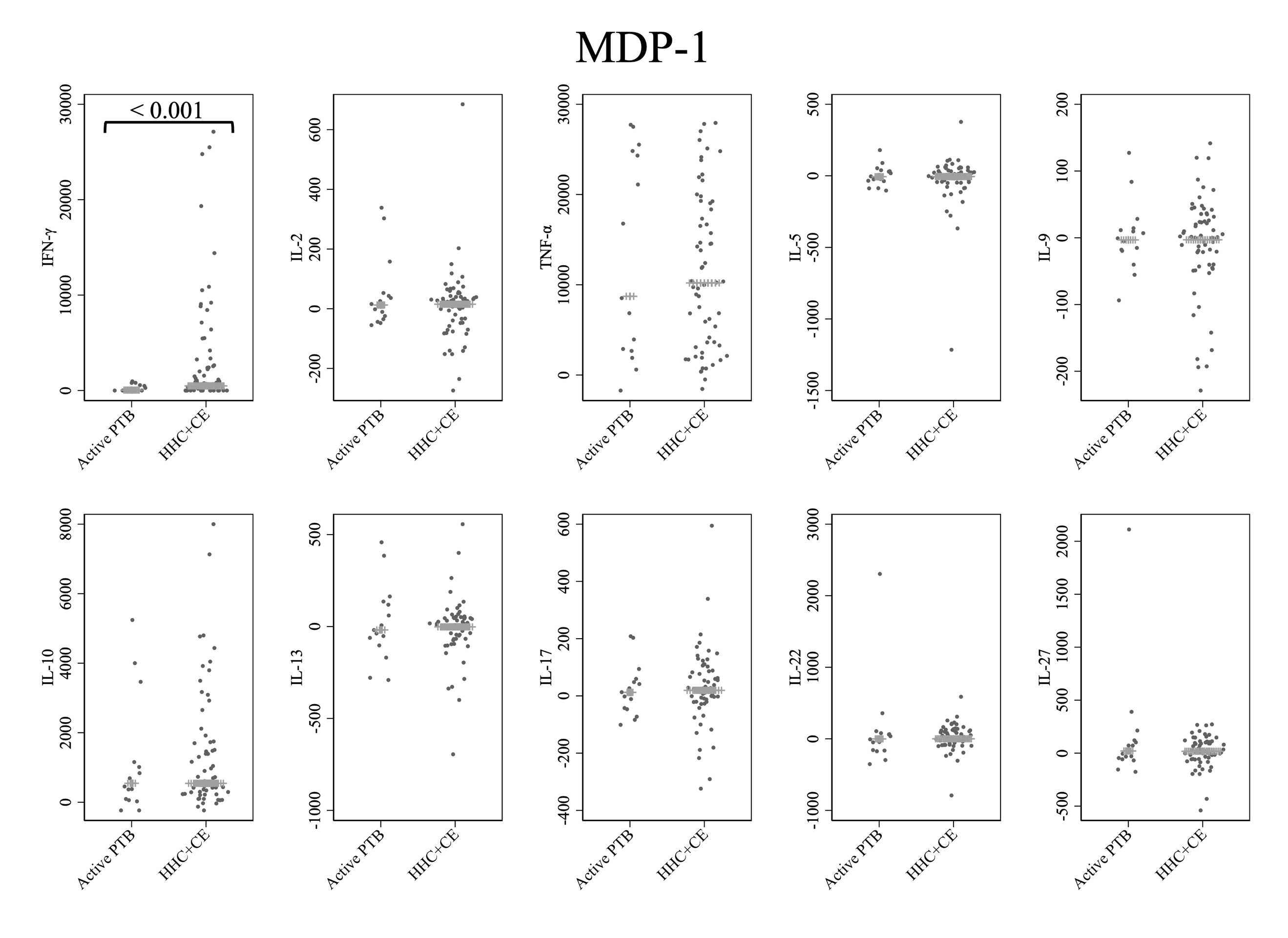


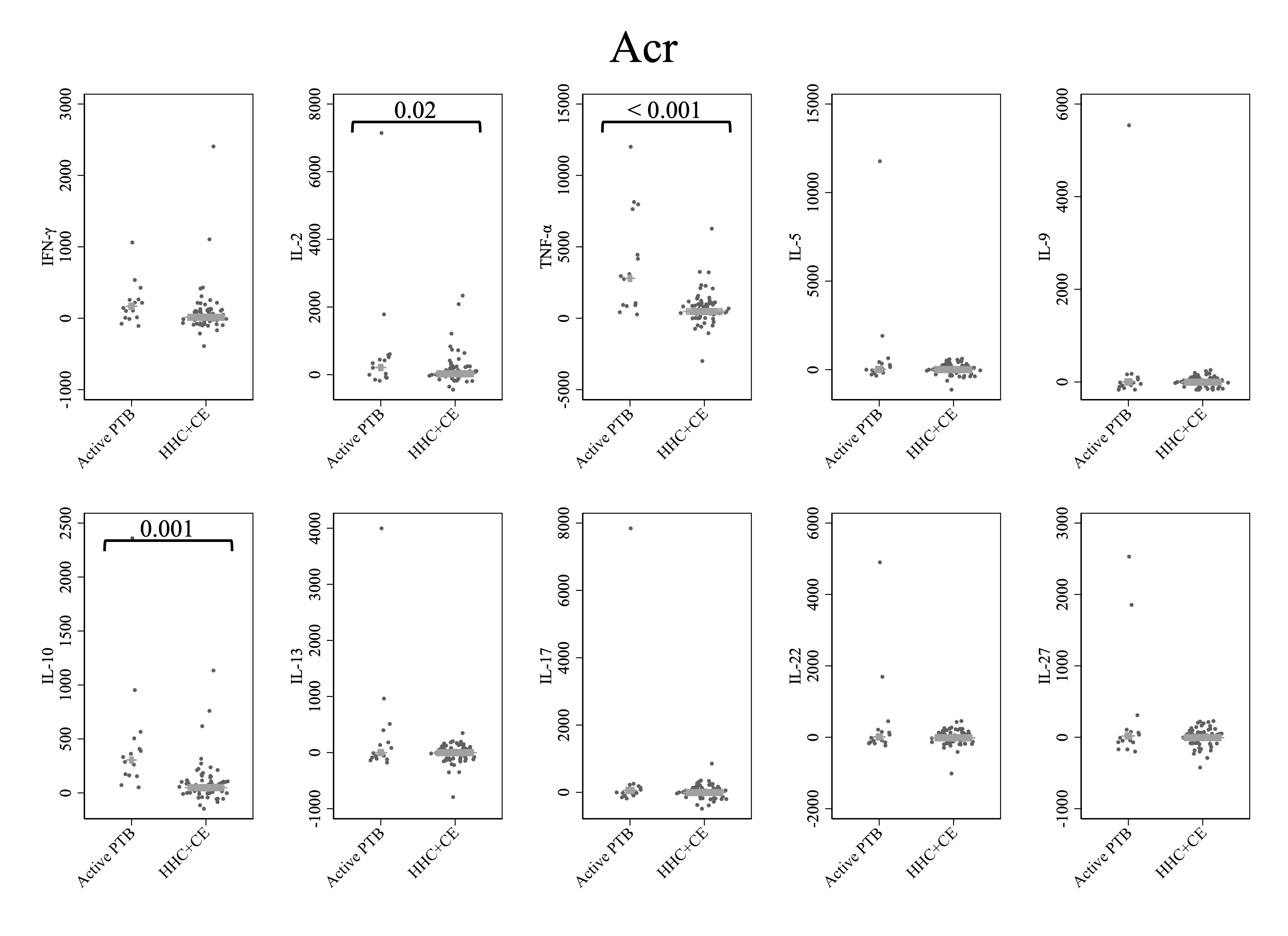


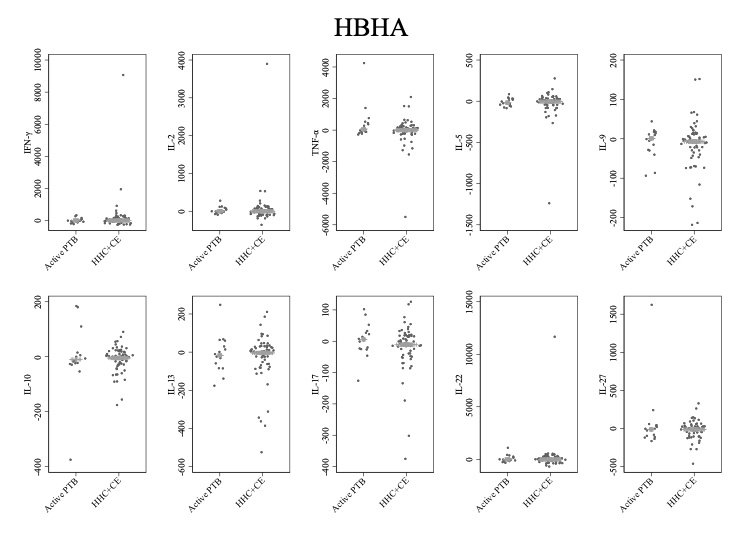


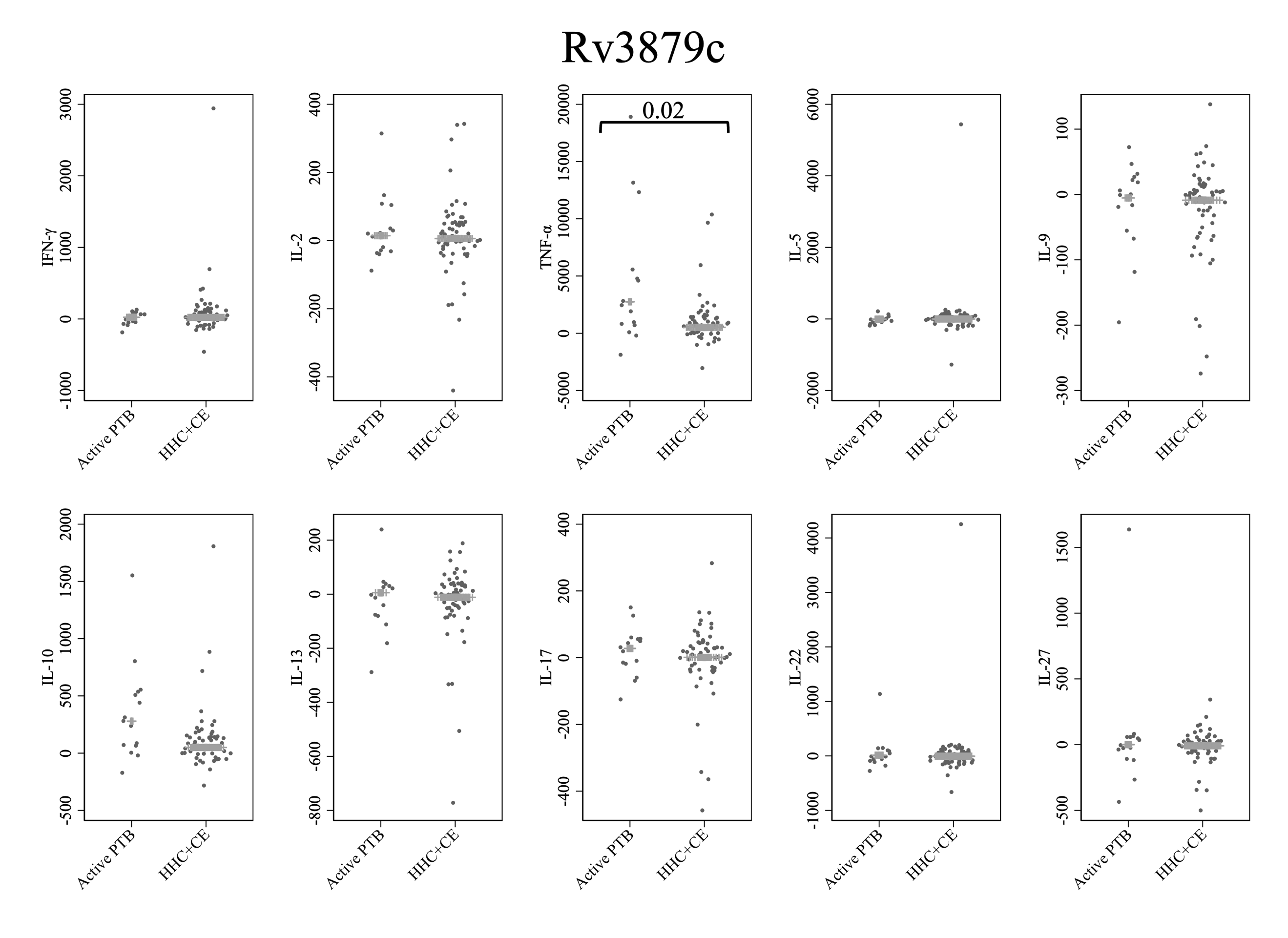


**Supplementary Figure 4.** Comparison between two groups: “active PTB” group versus group of “household contacts and community exposure subjects”.

The X-axis shows the participant groups. The Y-axis shows the concentrations of cytokines (pg/ml for IFN-γ, IL-2, TNF-α, IL-5, IL-9, IL-10, IL-13 and IL-17; ng/ml for IL-22 and IL-27). The horizontal lines show the medians. The numbers in the figure indicate adjusted p values. Comparisons of the concentrations of each cytokine between the “active PTB” group and the group of “household contacts and community exposure subjects” were performed using the Mann‒Whitney U test. We used linear regression to adjust for the potential confounders age, sex and BMI. Active PTB: active pulmonary tuberculosis, HHC: household contact, CE: community exposure.
